# In-silico insights on the prognostic potential of immune cell infiltration patterns in the breast lobular epithelium

**DOI:** 10.1038/srep33322

**Published:** 2016-09-23

**Authors:** J. C. L. Alfonso, N. S. Schaadt, R. Schönmeyer, N. Brieu, G. Forestier, C. Wemmert, F. Feuerhake, H. Hatzikirou

**Affiliations:** 1 Center for Information Services and High Performance Computing, Technische Universität Dresden, 01062 Dresden, Germany; 2 Braunschweig Integrated Centre of Systems Biology, Helmholtz Center for Infectious Research, 38124 Braunschweig, Germany; 3 Institute for Pathology, Hannover Medical School, 30625 Hannover, Germany; 4 Definiens AG, 80636 Munich, Germany; 5 Engineering Science, Computer Science and Imaging Laboratory, Université de Strasbourg, 67400 Strasbourg, France; 6 Modelling, Intelligence, Process and Systems, Université de Haute Alsace, 68093 Mulhouse, France; 7 Institute of Neuropathology, University Clinic Freiburg, 79117 Freiburg, Germany

## Abstract

Scattered inflammatory cells are commonly observed in mammary gland tissue, most likely in response to normal cell turnover by proliferation and apoptosis, or as part of immunosurveillance. In contrast, lymphocytic lobulitis (LLO) is a recurrent inflammation pattern, characterized by lymphoid cells infiltrating lobular structures, that has been associated with increased familial breast cancer risk and immune responses to clinically manifest cancer. The mechanisms and pathogenic implications related to the inflammatory microenvironment in breast tissue are still poorly understood. Currently, the definition of inflammation is mainly descriptive, not allowing a clear distinction of LLO from physiological immunological responses and its role in oncogenesis remains unclear. To gain insights into the prognostic potential of inflammation, we developed an agent-based model of immune and epithelial cell interactions in breast lobular epithelium. Physiological parameters were calibrated from breast tissue samples of women who underwent reduction mammoplasty due to orthopedic or cosmetic reasons. The model allowed to investigate the impact of menstrual cycle length and hormone status on inflammatory responses to cell turnover in the breast tissue. Our findings suggested that the immunological context, defined by the immune cell density, functional orientation and spatial distribution, contains prognostic information previously not captured by conventional diagnostic approaches.

Several studies provided conclusive evidence that a delicate balance between mammary epithelial cell proliferation and apoptosis regulates homeostasis in the healthy breast tissue^1^,^2^,^3^,^4^,^5^,^6^,^7^. After menarche, and in the absence of pregnancy, the adult female mammary gland is subjected to cyclic fluctuations depending on hormonal stimulation^1^,^8^. In response to such systemic hormonal changes, the breast epithelium undergoes a tightly regulated sequence of cell proliferation and apoptosis during each ovarian/menstrual cycle^1^,^2^,^3^. The peak of epithelial cell proliferation has been reported to occur during the luteal phase, suggesting a synergistic influence of steroid hormones, such as estrogen and progesterone^2^,^3^,^4^,^5^. In turn, the peak of apoptotic activity would be expected in response to decreasing hormone levels towards the end of the menstrual cycle^2^,^3^,^4^,^5^. However, recent histologic findings indicate that apoptosis reaches its maximum levels in the middle of the luteal phase, although there is also a peak at about the third day of the menstrual cycle^6^,^7^. Experimental measurements of cell turnover, i.e. programmed cell death and proliferation, demonstrated that an imbalance between the mitotic and apoptotic activity might lead to malignant transformation of epithelial cells and tumorigenic processes^9^,^10^,^11^. Indeed, excessive cell proliferation promotes accumulation of DNA damage due to insufficient timely repair and mutations^12^,^13^. There is also recent evidence that hormones suppress effective DNA repair and alter DNA damage response (DDR)^13^,^14^,^15^.

Previous models of transgenic mice engineered to develop mammary cancer demonstrated that abnormal patterns of cell turnover result in a higher risk of cancer development^16^. Moreover, genetic and epigenetic changes in genes that regulate mammary epithelial cell proliferation and apoptosis are considered possible initiators of breast carcinogenesis^17^,^18^. In fact, each cell in the human body faces everyday environmental challenges (e.g. ultraviolet light (UV) and terrestrial irradiation) that lead to DNA lesions that are constantly being repaired^19^. In addition to these exogenous agents, a mechanism particularly susceptible to DNA damage is DNA replication during cell division. Protection against DNA aberrations, arising via such physiological processes as DNA mismatches, is provided particularly by the *BRCA1*/*2* breast cancer susceptibility genes, which are crucial to avoid double-strand DNA damage during cell mitosis^20^,^21^. Mutations within *BRCA1* and *BRCA2* imply a high lifetime risk of developing carcinoma and account for most cases of familial breast cancers^21^,^22^. Experimental observations suggested that increased DNA damage levels and DNA repair defects are associated with an elevated risk of breast cancer^23^. Indeed, it is known that the development of tumors is associated with accumulation of DNA mutations in somatic cells^24^,^25^,^26^. Thus, mechanisms indicating failure to eliminate damaged epithelial cells may be equally promising candidates for novel breast cancer risk biomarkers as markers of DNA repair defects.

In the healthy breast tissue, lymphocytes are present and mainly localized within lobules rather than interlobular stroma^27^, with T-cells directly integrated in the lobular epithelium as part of the immune system (Figs 1 and 2 and Supplemental Fig. S5). There is strong evidence from murine models that immune cells carry out surveillance against infections and can eradicate nascent transformed cells before they grow into tumors^28^,^29^, indicating that the immune system plays a crucial role in maintaining the mammary gland function^27^,^30^. However, most of the factors and mechanisms that regulate the inflammatory microenvironment in the breast lobular epithelium are only partly understood^27^. In particular, those related to lymphocytic lobulitis (LLO), a characteristic pattern of inflammation frequently observed in non-neoplastic lobular structures adjacent to hereditary breast cancer, as well as in prophylactically removed breast tissue from patients without and with cancer-predisposing germ line aberrations *BRCA1*/*2*^27^,^31^,^32^,^33^,^34^,^35^. LLO has been defined based on estimates of immune cell number per lobule on hematoxylin and eosin (H&E) stainings. While some authors proposed a criterion of >100 lymphocytes per lobule^33^, others considered estimates of >50 lymphocytes per lobule for moderate and >100 lymphocytes per lobule for marked lobulitis^35^. However, the anatomical term lobule as a reference region for immune cell counting is prone to uncertainties due to variable shapes and sizes with respect to the number of epithelial cells^8^. Although LLO in non-neoplastic breast tissue has been recently associated with clinicopathologic features more commonly observed in hereditary breast cancer^33^,^35^, the biological and functional role of such inflammatory pattern in the normal lobular epithelium is unclear and the prognostic potential in the promotion of breast carcinomas remains to be elucidated. Figures 1 and 2 illustrate the full range of inflammatory patterns of CD8^+^, CD4^+^ and CD163^+^ cells observed within breast tissue from one individual healthy woman (Fig. 1) and across several healthy women (Fig. 2). Note the impressive heterogeneity within individuals and across different samples, and that even in the normal breast tissue, some lobules formally had to be assigned to LLO if any of the previous definitions would be consequently applied.

Accumulated experimental and clinical data indicate that the development of breast cancer is associated with a significant increase, and characteristic patterning, of both innate and adaptive immune cells^36^. In particular, T-lymphocytes and macrophages have been reported as the most abundant leukocytes present in neoplastic stroma. Furthermore, T-lymphocytes are also commonly associated with the development of breast cancer^36^, as well as macrophages that can influence growth, survival and invasion of tumor cells, angiogenesis and inhibition of cytotoxic T-cell responses which contributes to cancer immune evasion^37^. Additionally, during inflammatory responses certain types of macrophages produce a wide array of compounds that are likely to contribute to cancer initiation^38^. Previous findings led to the hypothesis that LLO could potentially be linked to pre-malignant precursor lesions or be associated with inherited breast cancer^27^,^33^,^35^. Indeed, the general functional relationship between inflammation and cancer has been extensively investigated^39^,^40^,^41^ and there is strong evidence from some cancer types that sustained cell proliferation in an environment rich in inflammatory immune cells and DNA damage promoting agents certainly potentiates neoplastic risk^24^,^26^,^41^,^42^. Considering these facts, the associated immune responses to few aberrant cells in breast tissue, that may give rise to malignancies due to impaired DNA-repair mechanisms by *BRCA1*/*2* mutations, may potentially be driving the earliest stages of cancer development. Therefore, a dynamic understanding of these early events is a crucial step towards investigating the prognostic role of inflammation in the breast lobular tissue.

For this purpose, we develop a cell-based model of epithelial and immune cell interactions in the breast lobular epithelium during the menstrual cycle. We use a novel approach that models immune system dynamics linked to the regular cyclic changes influenced by hormones of epithelial cell turnover in the breast tissue. The aim is to interpret *snapshot*-*like* single time points of breast tissue samples in the context of a mathematical model by considering crucial aspects of the underlying dynamic processes. Beyond the mechanistic understanding, we intend to generate insights into the functional and prognostic role of inflammation. Physiological model parameters are calibrated on the basis of data extracted from digital whole-slide images of immunohistochemical epithelial, vascular and immune cell markers on clinical annotation from a cohort of non-neoplastic women who underwent reduction mammoplasty due to orthopedic or cosmetic indications. Initially, we investigate recurrent inflammation during physiological menstrual cycles and normal hormone levels. Then, we systematically analyze parameter perturbations that may potentially lead to oncogenic events. In particular, we qualitatively analyze lobular inflammation patterns under systematic variation of model parameters, such as increased epithelial cell turnover rates influenced by different hormone levels, length of the menstrual cycle, impaired DNA-repair mechanisms due to *BRCA1*/*2* mutations and cytotoxic immune responses. Model results suggest that immune infiltrate spatial patterns and integration of personalized clinical data in the process of interpreting image-based findings could contribute to the extension of criteria for biopsy evaluation and development of novel prognostic markers.

## Material and Methods

### Experimental data

We consider breast tissue samples from a cohort of 22 healthy women, with ages ranging from 21 to 53 years (median age 30.5 years), who underwent reduction mammoplasty due to orthopedic or cosmetic indications^43^. The breast tissue samples were obtained during surgical procedures. Patients were not taking any oral contraceptives at the time of surgery. They were all premenopausal women without clinical abnormality nor familial history of breast cancer. Relevant co-morbidity, in particular autoimmune diseases including *type I diabetes mellitus (T1DM*), was not reported by any patient. Moreover, no clinical or radiological signs of breast lesions were reported in the clinical records. The phases of the menstrual cycle were determined by anamnestic documentation of the menses prior to the surgical intervention and complemented by asking the patients to report the date of onset of their first menstruation after surgery. Menstrual cycles range from 25 to 38 days, with 10 patients having a cycle length of 28 days (Supplemental Table S1). Patients were divided into luteal phase from 0–11 days between the surgery time and onset of menses afterwards, and into follicular phase from 17–30 days between both dates avoiding uncertainties during mid cycle (Supplemental Table S2). This dataset is used to calibrate the model parameters during physiological homeostasis.

**Figure 3:**
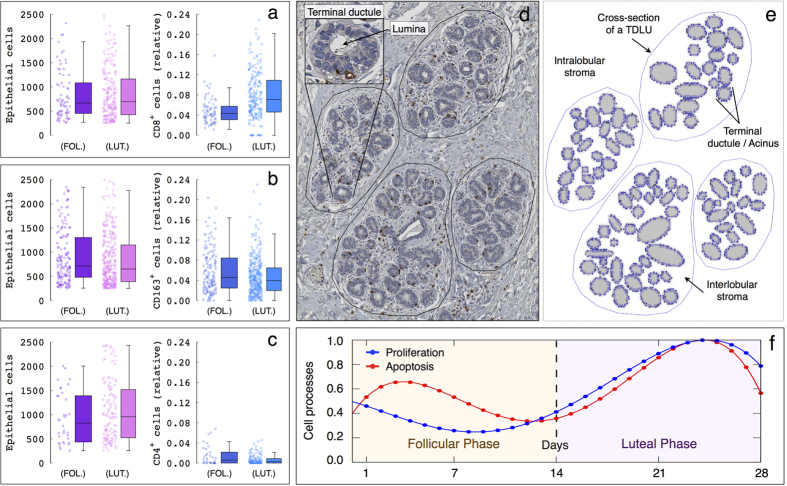
Experimental data, agent-based model simulation domain and epithelial cell turnover during the menstrual cycle. (**a**–**c**) Quantification of epithelial and immune cells in breast lobular tissue. From left to right, amount of epithelial cells and relative number of (**a**) CD8^+^, (**b**) CD163^+^ and (**c**) CD4^+^ cells with respect to the menstrual cycle phase in healthy women. Each box is drawn around the region between the first and third quartiles of the data points, with a horizontal line at the median value and whiskers extend for a range equal to 1.5 times the interquartile range. (**d**,**e**) Cross-sections of terminal ductal lobular units (TDLUs). (**d**) Annotated TDLUs composed by several terminal ductules/acini and (**e**) the corresponding simplified simulation domain, where luminal (light blue) and myoepithelial (dark blue) cells are represented, respectively. (**f**) Normalized curves of the epithelial cell proliferation (*PI*) and apoptosis (*AI*) indices with respect to the menstrual cycle. The follicular phase ranges from day 0 to 14, while the luteal phase between days 14 and 28.

The formalin-fixed and paraffin-embedded breast tissues were cut in sections of 3 *μm* thickness. Immunohistochemistry was performed to stain for cytotoxic T-lymphocytes (CD8; Dako M7103), regulatory T-cells (CD4; Zytomed Systems 503-3354) and macrophages (CD163; medac diagnostika 163M-16) on an automated instrument (Ventana Benchmark Ultra). Quantification of epithelial and immune cells was performed in about 650 (luteal phase) and 250 (follicular phase) representative breast lobular structures with between 250 and 2500 epithelial cells in whole slide images by means of an automated image analysis workflow. More precisely, a module for automated detection of single cells and nuclei from the Definiens Tissue Phenomics framework was used, which is based on the Definiens Image Miner and Developer Software^44^. The healthy women in our cohort were all adult at the time of the surgical procedure. Thus, we assumed that the adult breast is not growing and the epithelial compartment of mammary gland is only subject to a normal, overall balanced cell turnover/renewal at each menstrual cycle. In the following, the amount of immune cells is provided with respect to the number of epithelial cells and referred to as *relative number*. Figure 3a–c shows the resulting epithelial and immune cell quantification in normal breast tissue samples with respect to the follicular and luteal menstrual cycle phases (Supplemental Fig. S6).

We further consider a “proof-of-principle” dataset consisting of breast tissue samples obtained during surgical procedures from (i) 7 premenopausal healthy women who underwent reduction mammoplasty (RM) without clinical abnormality nor familial history of breast cancer, ages ranging from 21 to 45 years (median age 32 years), (ii) 21 premenopausal women who underwent prophylactic mastectomy (PM) due to an increased risk of hereditary breast cancer (*BRCA1/2* mutations) without clinical or pathological abnormalities, ages ranging from 22 to 53 years (median age 38 years), and (iii) a cohort of 7 premenopausal women with clinically manifest breast cancer, ages ranging from 30 to 43 years (median age 41 years). No details about the menstrual cycle phase were reported in the clinical records as it is not routinely collected. This serie of breast tissue samples is considered as a validation set of the model-based inflammatory pattern predictions.

The research was carried out in accordance with all relevant laws, guidelines and regulations. Experimental protocols including research use of surplus diagnostic materials were approved by the local institutional review board (Ethics Commission of Hannover Medical School; vote #2063-2013). Informed consent from patients was obtained from all subjects.

### A multiscale model of immune cell infiltration in breast lobular epithelium

We develop a multiscale agent-based model of epithelial and immune cell interactions in the breast lobular epithelium. Each cell is individually represented and its fate is determined by mechanistic rules and microenvironmental cues. In the following, we summarize the main model assumptions and experimental data-based parameterization (see the Supporting Information for further details).

#### Breast lobular epithelium geometry and cell types

The model accounts for myoepithelial and luminal cells that comprise the breast lobular epithelium. The human mammary gland constitutes a branching ductal-lobular system composed of an inner layer of polarized luminal cells and an outer layer of myoepithelial cells^8^. The luminal lineage contains polarized cells that line the lumina of mammary ducts which may contain secretory material. In turn, myoepithelial cells are localized between luminal cells and intralobular stroma, which positions them to communicate with both compartments^45^. These cellular layers are separated from the collagenous interstitial stroma by a basement membrane, which is not a limitation for immune cell movement (Supplemental Fig. S5)^8^,^46^. The breast stroma is made up of collagen and fibroblasts, interlobular dense fibro connective tissue, intralobular loose connective tissue and blood vessels^8^,^46^,^47^,^48^. This ductal network terminates in lobular units, commonly referred to as terminal duct lobular units (TDLUs), composed by lobules that are formed from tubular epithelial structures^8^,^46^.

Immune cells are crucially involved in maintaining the mammary gland function^30^,^49^. Lymphocytes are present and mainly localized to lobular structures rather than in the interlobular stroma, with T-cells directly integrated into the epithelium as part of the immune system (Supplemental Fig. S5f)^27^. Among the main immune components in the breast lobular tissue are the effector/regulatory T-cells and monocyte/macrophages^27^. In particular, CD4^+^ cells, CD163^+^ macrophages and CD8^+^ T-lymphocytes have been reported to be present in the breast tissue lobules with and without lobulitis (Supplemental Figs S8, S10 and S11)^27^, as well as involved in several cancer-related processes^50^. Cytotoxic CD8^+^ T-lymphocytes (CTLs) have the potential to kill several damaged cells by direct contact, indicating that they may recycle from one target to another and makes them very efficient effector cells^51^,^52^. It is known that (effector) CD8^+^ T-cells carry out their killing function by releasing cytotoxic proteins, which activates an endogenous apoptotic pathway within the target cells^53^,^54^. Besides many other functions, both CD4^+^ T-cells and CD163^+^ macrophages can suppress the cytotoxic responses of effectors resembling the function of regulatory cells^55^,^56^,^57^,^58^,^59^. The scavenger receptor CD163 is considered as a highly specific monocyte/macrophage marker for alternatively activated macrophages, often referred to as “M2” phenotype, with the potential to mediate anti-inflammatory effects^55^. These CD163^+^ myeloid derived cells could be suppressor cells that have been reported with an immunosuppressive effect on effector-dependent responses^55^,^56^,^57^. In turn, subsets of CD4^+^ T-helper cells have also the potential to suppress the cytotoxic responses of CD8^+^ T-cells, which is a key component of antitumor immunity^58^,^59^. Accordingly, we assume in a simplified strongly approach that, while *effector* CD8^+^ cells are the only responsible for killing (*target*) damaged epithelial cells, the function of *regulatory* CD4^+^ and CD163^+^ cells is a general suppression of effector-dependent responses by inducing inactivation.

The simulation domain represents cross-sections of terminal ductal lobular units (TDLUs) composed by several terminal ductules/acini, each with an inner layer of luminal cells surrounded by an exterior layer of myoepithelial cells. A cross-section of a TDLU is defined by a polygon delimiting the intra- and interlobular stroma (Fig. 3d,e). The separation between the inter- and intralobular stroma is based on stainings of Collagen IV (Supplemental Fig. S5), as well as on the annotations usually performed by pathologists during tissue biopsy evaluation. This clear separation between the lobular tissue and intralobular stroma is also supported by stainings of CD8, CD163 and CD4, where we observe that positive cells are mainly located close to the breast epithelium (Supplemental Figs S8, S10 and S11). In the mammary gland, as well as in other epithelial structures, cells are polarized into apical and basal cell compartments, connected by physical bonds such as tight junctions. The basal membrane keeps epithelial cells neatly organized in their functional structure, clearly separated from the underlying connective and fat tissue^8^,^46^. Accordingly, luminal and myoepithelial cells are assumed firmly placed within the breast lobular epithelium. On the contrary, immune cells are motile and can be located everywhere in the intra- and interlobular stroma except into the lobular lumina^8^,^46^.

#### Cell states

We assume that epithelial cells could become aberrantly damaged by DNA repair defects, for instance due to *BRCA1*/*2* mutations, that result in impaired DNA-double strand break repair mechanisms^21^,^20^. While epithelial cells dying by apoptosis (programmed death) would not pose any threat to the organism, damaged cells may represent a risk for breast cancer and need to be actively eliminated by the immune system. In the model, we assume that epithelial cells may be either in a normal, damaged or dying state. Moreover, *normally* dying apoptotic cells send out chemotactic *find*-*me* signals, which recruit innate immune cells to verify that apoptosis is correctly accomplished and to induce the clearance of the resultant debris^60^,^61^,^62^. These find-me signals create local gradients that serves to bring nearby immune cells to the dying cell^63^. In turn, *aberrantly* damaged cells that are not yet cancer cells, but have the potential of malignant transformation (see *hallmarks of cancer* concept^24^,^26^), are a source of chemotactic signals that attract and activate immune cells. Recent evidence indicates that the DDR signaling cascade influences the biology of the surrounding cellular microenvironment in a paracrine manner which induces an immune response^64^,^65^. This response to persistent DNA damage activates a variety of transcription factors that induce the expression of different immune genes including inflammatory cytokines and chemokines^64^,^66^,^67^. The damaged epithelial cells are seen as *refusing to die quietly* and need to be actively killed by effector cells before they can cause severe problems^24^,^26^. In particular, we assume that damaged and dying epithelial cells release two different chemokines (or chemotactic cytokines) inducing immune responses. Spatio-temporal dynamic of chemokine concentrations is modeled as a continuous field by a partial differential equation (see the Supporting Information for further details).

The chemotactic ability of cytokines is critical for the trafficking, homing and activation of leukocytes crucial to the innate immune response^68^. In fact, inflammatory chemokines are known as potent leukocyte activators expressed in inflamed tissues, and are therefore mostly considered as pro-inflammatory mediators^68^,^69^,^70^. We assume that immune cells become activated by chemokines from damaged epithelial cells. On the other hand, activated immune cells become inactive in the absence of such chemoattractant signals or due to the suppressing action of regulatory cells in the case of effectors.

#### Cell processes

**Figure 4:**
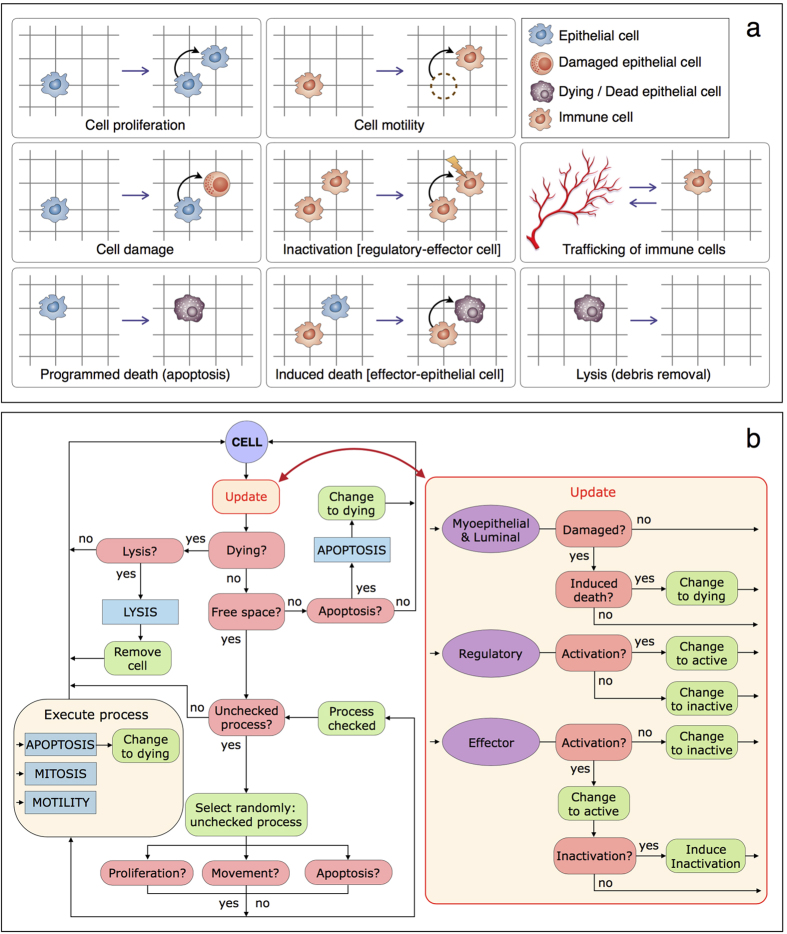
Agent-based model formulation details. (**a**) Schematic representation of the cell processes mimicked in the agent-based model. (**b**) Flowchart describing the checkpoints and possible actions that cells can perform in each simulation time-step.

Figure 4a shows a schematic representation of the cell processes mimicked in the agent-based model, i.e. proliferation of epithelial cells, immune cell motility and trafficking, death of damaged epithelial cells by effectors, inactivation of effectors by regulatory cells, programmed cell death (apoptosis) and lysis (removal of cellular debris). Cellular processes are represented in the model by stochastic events at different rates depending on the cell type and internal state.

- Immune cell trafficking. Blood vessels are essential for trafficking of immune cells^68^,^70^. The stroma of the mammary gland is made up of fat tissue, interlobular dense fibro connective tissue, intralobular loose connective tissue and blood vessels^8^,^46^,^47^,^48^. We assume that blood vessels are homogeneously distributed in the intra- and interlobular stroma (Supplemental Fig. S3). This assumption implies that immune cells can enter and leave the system from any node in the simulation domain.Lymphocyte traffic is mainly controlled by chemokines^68^,^69^,^70^,^71^. We assume that chemokines from damaged epithelial cells influence the trafficking of both regulatory and effector cells, while chemokines from dying epithelial cells only affect the trafficking of effector cells. The latter assumptions are based on our experimental observations. While in homeostasis the amount of (effector) CD8^+^ T-lymphocytes significantly change during the menstrual cycle, the number of (regulatory) CD4^+^ and CD163^+^ cells can be considered that remains almost invariant (Fig. 3a–c).
- Motility. Immune cell movement is only limited to the lumina of the acini (Fig. 3d,e). Once out of the bloodstream, immune cells follow chemokine gradients by a process called chemotaxis, which allows them to reach sites of inflammation and during normal homing to lymphoid organs^68^,^69^,^70^,^71^. We assume that immune cells are subject to chemotactic movement in response to chemokines from damaged and dying epithelial cells. On the other hand, immune cells are subject to random movement in the absence of chemoattractant signals.
- Immunosuppression. Activated regulatory cells permanently inactivate, by direct contact, effector cells at a certain rate. This rate increases proportionally with the number of activated regulatory cells surrounding an activated effector cell.
- Proliferation and Damage. Epithelial cells can divide at an intrinsic rate *k*_*pro*_, which results in a daughter cell that in turns can become aberrantly damaged at a rate *k*_*dge*_. Damaged epithelial cells can proliferate giving rise to an identical damaged daughter cell. Moreover, damaged epithelial cells never die due to apoptosis needing to be actively eliminated by effector cells, and produce a chemoattractant signal that induces the recruitment of immune cells. For simplicity, we assume that immune cells do not proliferate, and therefore their dynamic is exclusively characterized by motility and trafficking.
-  Programmed cell death. Undamaged epithelial cells are subject to apoptosis at an intrinsic rate *k*_*apt*_. Moreover, we assume that damaged epithelial cells can only die by the cytotoxic action of activated effector cells.
- Induced cell death. Activated effector cells induce death, by direct contact, to damaged epithelial cells at a rate *k*_*kill*_. This rate increases proportionally with the number of activated effectors surrounding a target epithelial cell.
- Lysis. Disposal of cellular debris resulting from apoptosis is carried out by a lysis process that removes dead epithelial cells from the model simulation domain.

#### Simulation flowchart and cell life cycle scheme

The flowchart in Fig. 4b shows how the accessible model states for the respective transition of a configuration of *N* cells are determined algorithmically in the simulations. Every cell includes a stochastic decision-making process that models phenotypic/state changes according to mechanistic rules and microenvironmental cues. At each simulation time-step, cells are randomly selected for which an additional process to update cell states is also randomly executed to avoid any order bias. In the case of aberrantly damaged epithelial cells, a checkpoint for induction of death by activated effector cells is executed. Moreover, immune cells have a checkpoint for activation and inactivation. Effector cells can be permanently inactivated by direct contact with activated regulatory cells. After this update process, dying epithelial cells are subject to a lysis process inducing its removal from the lattice. Otherwise, undamaged epithelial cells in a node with no free neighbor nodes can die due to apoptosis. If there is a free node next to the selected cell, processes such as motility, proliferation and apoptosis are randomly selected until one is executed or all of them have been verified but not triggered. If motility is executed, the cell moves to a free neighbor node, while proliferation produces a daughter cell. This iterative process is executed until all cells have been updated in the current simulation time-step. At the end of each simulation time-step, immune cell populations are updated through an iterative process. In particular, immune cell trafficking is governed by rates depending on the intensity of chemokines from damaged and dying epithelial cells (see the Supporting Information for further details).

#### Parameter calibration

Assessments of epithelial cell proliferation and apoptosis in healthy human mammary tissue during the follicular and luteal phase of the menstrual cycle have been recently reported^2^,^3^,^4^,^7^. In particular, estimates of a proliferation (*PI*) and apoptotic (*AI*) index, defined as the number of Ki-67 and TUNEL positive cells per 1000 epithelial cells, were provided^7^. The highest *PI* value was detected near the end of the menstrual cycle, whereas the *AI* reached its maximum at the beginning and end of the menstrual cycle. The *PI* values were estimated between 30.46 (luteal phase) and 13.45 (follicular phase). The cell renewal index *PI*/*AI* was also significantly higher in the luteal phase of the menstrual cycle.

**Figure 5:**
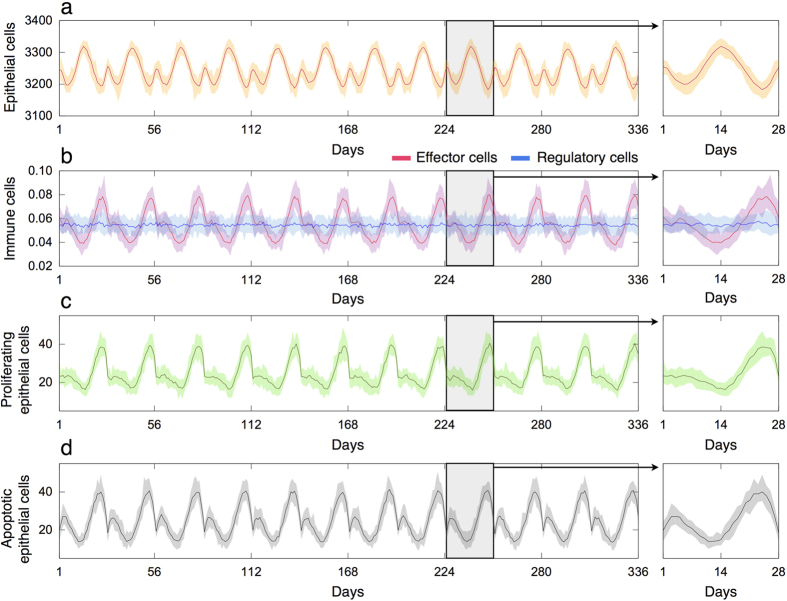
Time evolution of epithelial and immune cells in healthy breast tissue. The results are averaged over 20 simulations each consisting in 12 menstrual cycles of 28 days and normal hormone levels. (**a**) Amount of epithelial cells. (**b**) Relative number of regulatory and effector cells. (**c**,**d**) Number of proliferating and apoptotic epithelial cells per 1000 epithelial cells, respectively. Simulation results for a single menstrual cycle are represented in the right panels.

We consider normalized versions of the *PI* and *AI* curves with respect to the menstrual cycle^7^ to modulate the intrinsic proliferation *k*_*pro*_ and apoptotic *k*_*apt*_ rates of epithelial cells (Fig. 3f). This allows to reproduce the oscillations of cell turnover in the lobular epithelium during menstrual cycles and normal hormone levels. Assuming the physiological plausible proliferation rate of epithelial cells *k*_*pro*_ = 0.03 h^−1 ^72,73,74 and estimating the apoptotic rate as *k*_*apt*_ = 0.0021 h^−1^, we can reproduce the *PI* = 30.5 ± 22 values measured experimentally during the menstrual cycle (Fig. 5). We remark that the interdependent mitotic and apoptotic events should be balanced in adult women to keep breast tissue homeostasis. However, TUNEL-positive cells only represent a small part of the total number of apoptotic cells, since only the final phases within the time course of apoptosis are labeled. The same holds true for other apoptosis markers such as cleaved caspase-3, a marker for a short part of the execution phase of apoptosis. Indeed, only few cells were stained by cleaved caspase-3 marker (Supplemental Fig. S5), and their overall number was lower than the amount of (mitotic) Ki-67 positive cells. This is due to the fact that only short phases within the time course of apoptosis are labeled, whereas Ki-67 stains all cycle phases except the resting cells in G0 phase. Thus, the amount of apoptotic epithelial cells is calibrated to balance the *PI* values experimentally quantified.

Physiological parameters related to immune cell trafficking are calibrated based on the experimental data in Fig. 3a–c. Figure 5 shows the resulting time evolution of epithelial and immune cells in healthy breast tissue during several menstrual cycles. The mean relative number of effector cells in the follicular (day 1 to 11), in-between (days 11 to 17) and luteal (days 17 to 28) phases are 0.05 ± 0.01, 0.04 ± 0.01 and 0.07 ± 0.02, respectively. In turn, the mean relative number of regulatory cells during the menstrual cycle is 0.055 ± 0.01. The calibrated model parameters also provide accurate estimates of the relative number and spatial distribution of immune cells during the menstrual cycle (Supplemental Figs S6 and S7). We provide details in the Supporting Information about the immune cell trafficking implementation and estimation of the other model parameters.

## Results

Due to the stochastic nature of the model, we performed for each parameter constellation 10 simulations each consisting of 12 menstrual cycles (about 1 year) and monitored the average statistics. The relative number of damaged epithelial and immune cells are provided with respect to the total number of epithelial cells in the simulated cross-sections of TDLUs.

### Existence of a critical damage rate of epithelial cells that determines different inflammatory regimes

**Figure 6:**
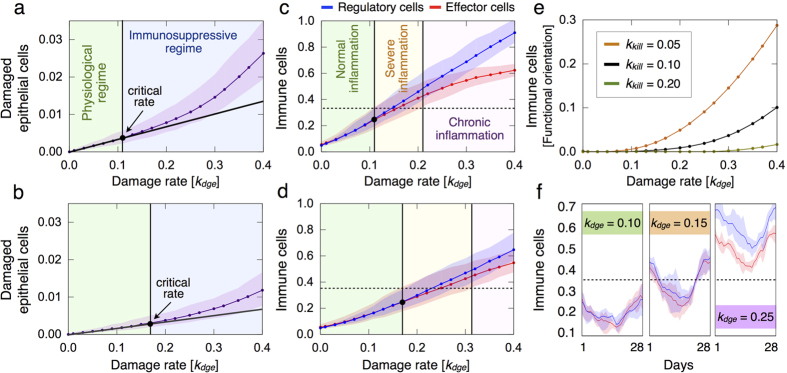
Inflammatory responses induced by increasing damage rates of epithelial cells. The results are averaged over 10 simulations, for every parameter constellation, each consisting in 12 menstrual cycles of 28 days and normal hormone levels. The mean (marked solid line) and min/max (shadow) values are represented. (**a**,**b**) Relative number of damaged epithelial cells for *k*_*kill*_ = 0.05 and *k*_*kill*_ = 0.10, respectively. (**c**,**d**) Relative number of immune cells for *k*_*kill*_ = 0.05 and *k*_*kill*_ = 0.10, respectively. (**e**) Difference between the relative number of regulatory and effector cells for different *k*_*kill*_ values. (**f**) Relative number of immune cells during a single menstrual cycle for *k*_*kill*_ = 0.05 and different *k*_*dge*_ values.

We first investigate the inflammatory responses to different damage rates of epithelial cells *k*_*dge*_, a menstrual cycle of 28 days and normal hormone levels. Figure 6a–d shows the resulting epithelial damage and immune responses to increasing *k*_*dge*_ values for different killing rates of damaged epithelial cells by effectors *k*_*kill*_. Model simulations predict that there exists a critical *k*_*dge*_ value from which the relative number of damaged epithelial cells increases non-linearly (Fig. 6a,b) and suppression of effector cell-dependent responses occurs (Fig. 6c,d). Below this critical threshold, epithelial damage scales linearly with *k*_*dge*_, which is associated with a tightly controlled inflammation (Fig. 6a,b). On the other hand, higher *k*_*dge*_ values result in stronger inflammatory responses with marked immunosuppression and epithelial cell damage. This may increase the risk of developing various breast pathologies, which may lead to oncogenic events. Figure 6a,b shows that such critical threshold depends on the effectiveness of the immune system to control epithelial cell abnormalities. When decreasing the killing rate *k*_*kill*_, the relative number of damaged epithelial and immune cells increases, and the resulting inflammatory responses exhibit stronger suppressive activity of regulatory cells to prevent pathological self-reactivity (compare Fig. 6c to 6d). Moreover, a decreased ability of effectors to kill target cells is predicted to contribute to immunological context heterogeneity in terms of the functional orientation of immune infiltrates (Fig. 6e). This suggests that not only the number of immune infiltrates, but also the functional orientation of the immune contexture, may be considered as a marker of epithelial damage.

Figure 6c,d shows high variations in the amount of immune infiltrates during the menstrual cycle when epithelial cell damage is triggered, which may have a significant impact on tissue homeostasis. In particular, we assume that inflammatory responses are physiological as long as the amount of damaged epithelial cells increases linearly with *k*_*dge*_ (Fig. 6a,b). Working under this assumption, the maximum relative number of immune cells given by such critical damage rate, which separates physiological from pathological responses, allows to characterize at least two other inflammatory regimes. One regime of severe inflammations for intermediate damage rates and the other regime of chronic (persistent) inflammations for higher *k*_*dge*_ values (Fig. 6c,d). While during chronic inflammatory responses immune cells never leave the breast lobular epithelium, for intermediate *k*_*dge*_ values the resultant inflammation resolves. Figure 6f shows that a higher amount of infiltrating immune cells is more frequent at the beginning (days 0–7) and end (days 21–28) of the menstrual cycle than at the middle (days 7–21). Moreover, we found that for a range of *k*_*dge*_ values, defining the regime of severe inflammations, there may be peaks of immune infiltrates closely resembling physiological and pathological conditions during the menstrual cycle (Fig. 6f). This leads to misleading normal inflammatory responses that need to be recognized. These findings suggest that inflammatory responses are oscillatory phenomena that depend on the epithelial cell damage rate and menstrual cycle length.

### Increased hormone levels enhance epithelial damage and inflammatory responses

**Figure 7:**
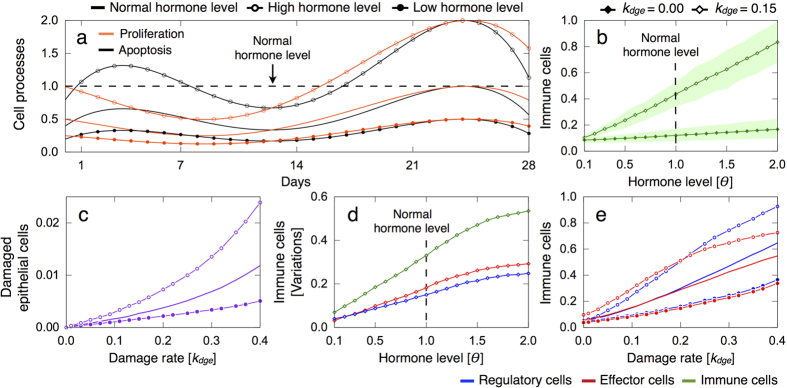
Influence of hormone levels on epithelial damage and inflammatory responses. The results are averaged over 10 simulations, for every parameter constellation, each consisting in 12 menstrual cycles of 28 days. The mean (marked solid line) and min/max (shadow) values are represented. (**a**) Normalized curves of epithelial cell proliferation and apoptosis during the menstrual cycle for normal (*θ* = 1.0), increased (*θ* = 2.0) and decreased (*θ* = 0.5) hormone levels. (**b**) Relative number of immune cells for increasing hormone levels and different *k*_*dge*_ values with *k*_*kill*_ = 0.10. (**c**) Difference between the maximum and minimum relative number of regulatory and effector cells, as well as the total amount of immune cells, for *k*_*dge*_ = 0.15 and *k*_*kill*_ = 0.10. (**d**,**e**) Relative number of damaged epithelial and immune cells for increasing *k*_*dge*_ values and different hormone levels with *k*_*kill*_ = 0.10, respectively.

To gain insights into the effect of hormonal changes on breast tissue damage and inflammatory responses, we modulate the normalized curves of epithelial cell proliferation and apoptosis during the menstrual cycle (Fig. 3f) by a positive scaling factor *θ*, i.e. referred to as the hormone level. By doing this, we assume that the hormonal status directly affects the cell turnover rate in the lobular epithelium^1^,^2^,^3^,^20^. Figure 7a shows different hormone-modulated sets of the normalized proliferation and apoptosis curves with respect to the menstrual cycle for *θ* = 0.05 and *θ* = 2.0. These values respectively correspond to decreased and increased hormone levels with respect to the normal status *θ* = 1.0.

Figure 7b shows that the relative number of immune cells in the breast lobular epithelium is predicted to increase linearly with rising hormone levels *θ*. We also found that irrespective of the hormonal status, when epithelial cell damage is triggered the amount of immune infiltrates significantly increases compared to physiological conditions. This is particularly critical for high *θ* values, which strongly influence the epithelial cell turnover rate and give rise to large fluctuations in the associated inflammatory responses. Figure 7c shows that epithelial damage increases in more enriched hormonal milieu, as well as variations in the amount of immune infiltrates in the lobular tissue during the menstrual cycle (Fig. 7d). In turn, the suppressive action of regulatory cells to effector responses is more prominent in breast tissues with high hormone levels (Fig. 7e).

### Epithelial damage induces clustering patterns of immune cells

**Figure 8:**
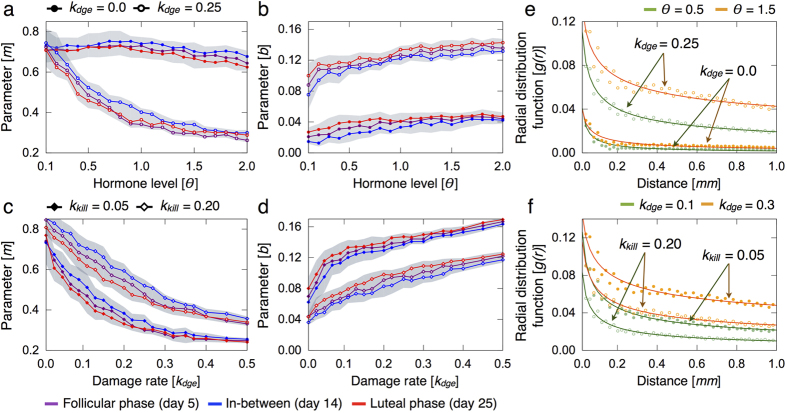
Influence of hormone levels and epithelial damage on the spatial distribution of immune cells. The results are averaged over 10 simulations for every parameter constellation. The mean (marked solid line) and min/max (shadow) fitted values are represented. (**a**–**d**) Estimates of parameters *m* and *b* of the power law *b* · *r*^−*m*^ that better fit the radial distribution functions *g*(*r*) at the follicular (day 5) and luteal (day 25) phase of the menstrual cycle, as well as in the middle (day 14). (**e**,**f**) Radial distribution functions *g*(*r*) at day 25 of the menstrual cycle for different parameter sets and the resulting power law fittings.

Spatial distribution of immune cells is determined by the radial distribution function *g*(*r*) that describes how density varies as a function of the distance from a reference immune cell *r*^75^. The function *g*(*r*) is calculated at the follicular (day 5) and luteal (day 25) phase of the menstrual cycle, as well as in the middle (day 14). We then consider the mean *g*(*r*) values over 10 simulations and fit them to the power law *g*(*r*) ≈ *b *· *r*^−*m*^. The parameter *m* represents the decreasing slope of *g*(*r*) and *b* the probability to find a neighbor immune cell, i.e. in direct contact. Figure 8 shows estimates of the spatial distribution of immune infiltrates in the lobular epithelium for different damage rates of epithelial cells *k*_*dge*_, hormone levels *θ* and effector killing rates *k*_*kill*_.

Figure 8a,b shows that in normal breast tissue lobules without epithelial damage, sparse and uniform spatial distributions of immune infiltrates are predicted irrespective of the hormone levels. The high and almost invariant values of *m* for increasing hormone levels indicate that immune cells are well distributed in the whole lobular epithelium (Fig. 8a). In turn, the low *b* values evidence that immune cell clusters are rare spatial patterns in normal breast tissue (Fig. 8b). On the other hand, model simulations suggest that epithelial damage induces clustering patterns of immune cells (Fig. 8a,b). Moreover, immune cell clusters are more frequent for elevated hormone levels as indicated by the decreasing *m* values and the high estimates of *b*. Figure 8c,d shows that cluster formation is favoured by high damage rates of epithelial cells *k*_*dge*_ and low effector killing rates *k*_*kill*_. Furthermore, model analysis reveals that the number of clusters is associated with the degree of epithelial damage and cluster sizes with the effectiveness of the immune response. Figure 8e,f shows the power law fittings of some radial distribution functions for different parameter combinations.

**Figure 9:**
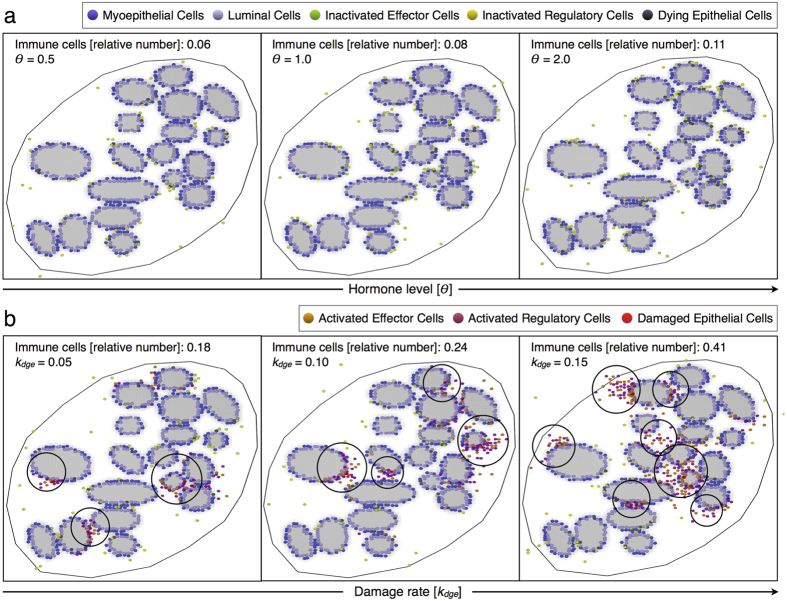
Immunological context in cross-sections of a terminal ductal lobular unit (TDLU). Relative number and spatial distribution of immune cells for (**a**) increasing hormone levels *θ* without epithelial damage *k*_*dge*_ = 0.0 and (**b**) increasing *k*_*dge*_ values and normal hormone levels *θ* = 1.0, with *k*_*kill*_ = 0.05.

**Figure 10:**
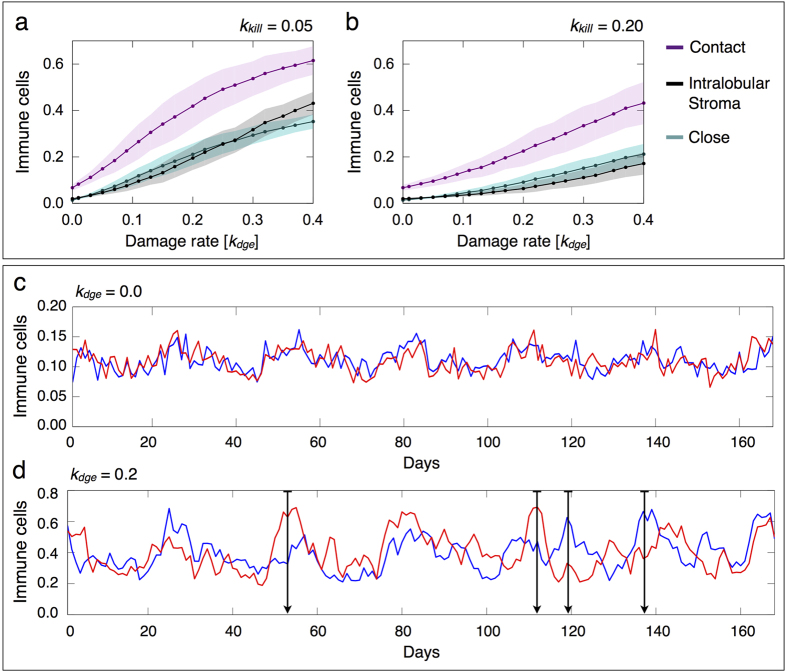
Spatial distribution of immune cells for increasing epithelial damage rates and time evolution of immune cells in cross-sections of terminal ductal lobular units (TDLUs). (**a**,**b**) The results are averaged over 10 simulations, for every parameter constellation, each consisting in 12 menstrual cycles of 28 days and normal hormone levels. The mean (marked solid line) and min/max (shadow) values are represented. Mean relative number of immune cells with respect to their position in the lobular epithelium for (**a**) *k*_*kill*_ = 0.05 and (**b**) *k*_*kill*_ = 0.02. (**c**,**d**) Simulations of 6 menstrual cycles each of 28 days and normal hormone levels. Relative number of immune cells in different TDLUs for (**c**) *k*_*dge*_ = 0.0 and (**d**) *k*_*dge*_ = 0.2, with *k*_*kill*_ = 0.02.

Figure 9 shows the relative number and spatial distribution of immune cells in a cross-section of a TDLU with and without epithelial damage. While immune cells are well distributed in the normal lobular epithelium irrespective of the hormonal status (Fig. 9a), clusters emerge broadly distributed for rising damage rates of epithelial cells (Fig. 9b). Model simulations predict that clustering patterns of immune cells are most common in the luteal phase of the menstrual cycle (Fig. 8). Moreover, we found that cluster formation is a faster process than resolution via dispersal of immune cells after killing the damaged epithelial cells. Model results support that immune cells are mainly localized in contact or close to epithelial cells, which is predicted to be more pronounced with epithelial damage irrespective of the *k*_*kill*_ value (Fig. 10a,b). However, large clusters of immune cells do not necessarily imply a more effective clearance of damaged cells, since most of the effectors may not be in direct contact with target cells (Fig. 9b).

### Epithelial damage results in different inflammatory patterns between TDLUs

We extend the model to the investigation of single TDLUs to reflect the heterogeneity of mammary tissue, where focal inflammatory changes can be close to unaffected areas. This type of heterogeneity not only in the density and functional orientation of immune infiltrates, but also in the inflammation patterns between close lobular structures, is suggested relevant for a more comprehensive picture of the epithelial damage in a realistic clinical context. Figure 10c,d shows the time evolution of the relative number of immune cells in different cross-sections of TDLUs with and without epithelial damage. We found similar degrees of inflammation between different healthy TDLUs irrespective of the menstrual cycle phase (Fig. 10c). However, epithelial damage results in different amounts of immune infiltrates across lobular structures in their spatial context, assuming that these *neighbours* have the same intrinsic cell features (Fig. 10d). Although this result is obtained mainly in the luteal phase of the menstrual cycle, it can also be observed in the follicular phase. This extends our previous findings in Fig. 9, where we indicated that inflammation patterns are spatially heterogeneous within TDLUs for selected time points. Here we show that relevant heterogeneity also exists between lobular structures. Therefore, quantifying the degree of inflammation in several TDLUs and at distinct time points is predicted to provide a better net estimation of the immunological context and breast tissue damage.

### Shorter menstrual cycles enhance epithelial damage and persistence of inflammation

**Figure 11:**
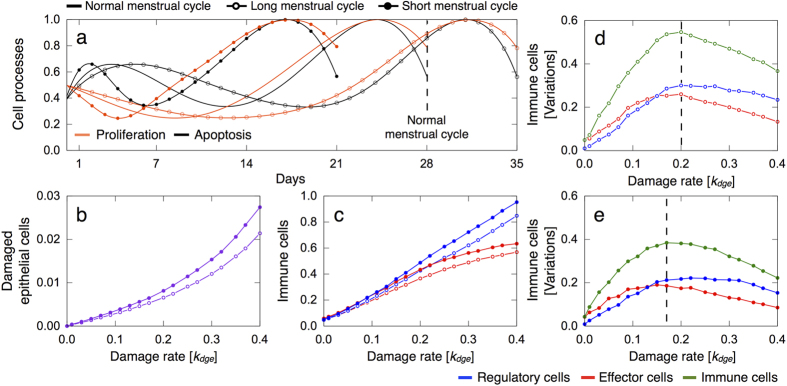
Influence of menstrual cycle length on epithelial damage and inflammatory responses. The results are averaged over 10 simulations, for every parameter constellation, each consisting in 12 menstrual cycles and normal hormone levels. (**a**) Normalized curves of epithelial cell proliferation and apoptosis for menstrual cycles with the follicular phase of 7, 14 and 21 days, with the luteal phase 14 days long. (**b**,**c**) Mean relative number of damaged epithelial and immune cells in the short and long menstrual cycle for increasing *k*_*dge*_ values. (**d**,**e**) Difference between the maximum and minimum relative number of regulatory and effector cells, as well as the total amount of immune cells, in the short and long menstrual cycle for increasing *k*_*dge*_ values.

Epidemiologic studies have reported that the luteal phase is consistently 14 days long with only minor variations, whereas the duration of the follicular phase usually ranges from 10 to 21 days^48^. This is largely in line with the distribution of total menstrual cycle length (follicular and luteal phases) in the cohort of the patients considered^43^, which varies from 25 to 38 days (Supplemental Table S1). We investigate the role of menstrual cycle length on epithelial damage and inflammatory responses by simulating follicular phases of 7, 14 and 21 days, with the luteal phase 14 days long. This results in a short, normal and long menstrual cycle of 21, 28 and 35 days, respectively. To do that, we dilate and contract the normalized curves of epithelial cell proliferation and apoptosis in the follicular phase 0 ≤ *t* ≤ 14, while keeping invariant the curve shapes in the luteal phase (Fig. 11a).

The menstrual cycle length is predicted to be an important factor influencing the inflammation degree. Fig. 11b shows that, in general, short menstrual cycles correlate with an increased amount of damaged epithelial cells rather than longer periods. This is due to the major relative contribution of the luteal phase, where the probability of epithelial damage is higher because increased cell turnover rates. In general, one could assume that a longer follicular phase may give to the body more *relaxation time* after a phase of intense cell turnover and, as a consequence, favoring cytotoxic effector responses. In fact, Fig. 11c shows that short menstrual cycles induce stronger inflammations in response to increasing *k*_*dge*_ values. Model simulations predict that irrespective of the follicular phase length, there exists a critical *k*_*dge*_ value for which variations in the amount of immune infiltrates in the lobular tissue during the menstrual cycle first increase and then decrease again. Comparing Fig. 11d to Fig. 11e, we observe that such variations significantly increase for longer periods. This implies that for high damage rates of epithelial cells, shorter menstrual cycles lead to more severe and persistent inflammatory responses.

## Discussion

In this work, we investigated the prognostic potential of inflammatory patterns in non-malignant breast lobular tissue in the context of related pathologies. The accurate interpretation of inflammatory patterns observed in diagnostic biopsies in the context of breast cancer requires a deeper understanding of the range of normal immune cell infiltrations in the course of the menstrual cycle, as well as a better definition of how physiological states can be differentiated from potentially pathological conditions such as lymphocytic lobulitis (LLO). To that end, we developed an agent-based model of immune and epithelial cell interactions in the breast lobular epithelium under the influence of hormonal changes during the menstrual cycle. Physiological model parameters were calibrated on the basis of spatial data extracted from digital whole-slide images of immunohistochemical epithelial, vascular and immune cell markers. These images were acquired on clinical annotations from a cohort of healthy patients who underwent reduction mammoplasty^43^. Reduction mammaplasty tissues can be regarded as the best approximation to the normal state, as close as possible to the physiological situation. The patients do not have a specific risk, nor an established cancer, and one can assume a steady state between proliferation and apoptosis in the adult irrespective of the breast size. This is confirmed by recent data comparing reduction mammoplasty tissues to an exceptional series of small needle biopsies of healthy volunteers, reporting no major differences besides a slightly increased incidence of non-malignant “proliferative disease” (focal benign changes without cancer risk), but no evidence for disturbed overall cell turnover^76^. To better align the model parameters with microscopic observations, we used a modular workflow combining a convolutional neural network to detect regions of interest with an auto-adaptive random forest pixel-wise classifier to detect nuclei (*nucleus container* module; Definiens, Germany). This analysis of immune cell density and location provided the quantitative read-out required as input data of the proposed agent-based model. The immunological context as defined by the density, functional orientation and spatial distribution of immune cells, is evidenced to provide valuable information beyond currently used descriptive categories to predict potentially pathological scenarios.

Biopsies are single tissue samples typically extracted for a diagnostic purpose, which are by definition always performed at a certain time-point that is mostly determined by clinical and organizational needs. In contrast to slowly developing pathological changes of epithelial structures, such as benign lesions (e.g. hyperplasia, metaplasia, cysts), pre-malignant lesions (e.g. proliferative disease with atypia), and malignant neoplasia (e.g. carcinoma *in situ* or invasive carcinoma), inflammatory changes are prone to temporal variations. Other than stationary epithelial lesions, inflammatory patterns are composed of motile cells that can change their relative location, differentiation and activation status within few days (or even hours) in response to microenvironmental cues. Our approach accounts for this particular situation by introducing a temporal component into the concept of biopsy evaluation. In particular, we propose to interpret the *snapshot*-*like* single time-point of a tissue sample in the context of a mathematical model representing central aspects of the underlying dynamic processes.

The clinical need for this kind of approach is a consequence of the rapid development of *oncoimmunology*, a therapeutic strategy to target the immune system of cancer patients with immunomodulatory compounds. Currently, several such therapies are in clinical testing in breast cancer, as well as in other tumor types. As a consequence, immune cell evaluation is shifted into the focus of diagnostic biopsy analysis^77^. So far, inflammatory changes such as LLO have largely been considered as *bystander*-*effects* in breast pathology, and historically the main focus of diagnostic evaluation used to be on the epithelial neoplasia^78^. This is changing dramatically. Meanwhile, there is even standardized protocol for *immunoscoring* in breast cancer^79^, however, this does not yet consider the inflammatory microenvironment of the non-malignant (pre-existing) mammary gland structures. We expect that immunological responses to the non-malignant epithelium provide valuable information on oncogenesis (i.e. the process leading to malignant transformation), and prognosis once a tumor is established. In this clinical context, our model predictions hint towards novel criteria for breast biopsy evaluation that allow for a more comprehensive analysis and potentially further prognostic parameters in breast cancer pathology. In particular, model results suggest that for immune cell evaluation (i) the time-point and eventually the optimal frequency of biopsy sampling should be considered, (ii) clinical patient data such as the hormonal status and the menstrual cycle phase should be taken into account and (iii) inflammatory events can be heterogeneous, indicating that the size and number of biopsies should be adjusted to the diagnostic purpose.

A first recommendation is based on the finding that the time point of a biopsy is critical for the density of immune infiltrates and inflammatory patterns. Model results suggest that inflammation is an oscillatory phenomenon during the menstrual cycle. This predicts that taking a breast biopsy sample at an arbitrary time point of the menstrual cycle could lead to wrong conclusions about the mammary gland inflammatory profile. Therefore, we propose that requests for breast tissue biopsy evaluation should always include information of the probable cycle phase and relevant medication (e.g. anticonception, anti-hormonal therapy, immunosuppressive drugs like corticosteroids, etc.). Another important result of our study is that changes in hormone levels strongly influence the amount of infiltrating lymphocytes present in the breast lobular tissue during the menstrual cycle. This touches aspects of a long standing discussion on whether or not oral anticonception can increase the risk of malignancies. So far, the stimulatory effects of hormone level modulations on epithelial cell turnover have been in the focus of the scientific discussion^2^,^3^,^14^,^46^,^48^,^80^. Model simulations show that the timing of hormonal cycles (e.g. the artificial induction of a 28-days-long menstrual cycle by oral anticonceptives), could induce an immunological phenomenon that potentially could link breast cancer risk to hormonal therapies. Moreover, model results suggest that the known effects of the synthetic steroid hormones on increased turnover rate of epithelial cells could be aggravated by the artificial limitation of the follicular phases to approximately 14 days. Following this line of reasoning, one could consider the variable, sometimes extended follicular phase in natural menstrual cycles as an immunological *recovery phase. Normalizing* or shortening this phase may enhance epithelial cell damage and persistent inflammation responses, hypothetically even leading to an increased risk of developing pre-neoplastic lesions.

**Figure 12:**
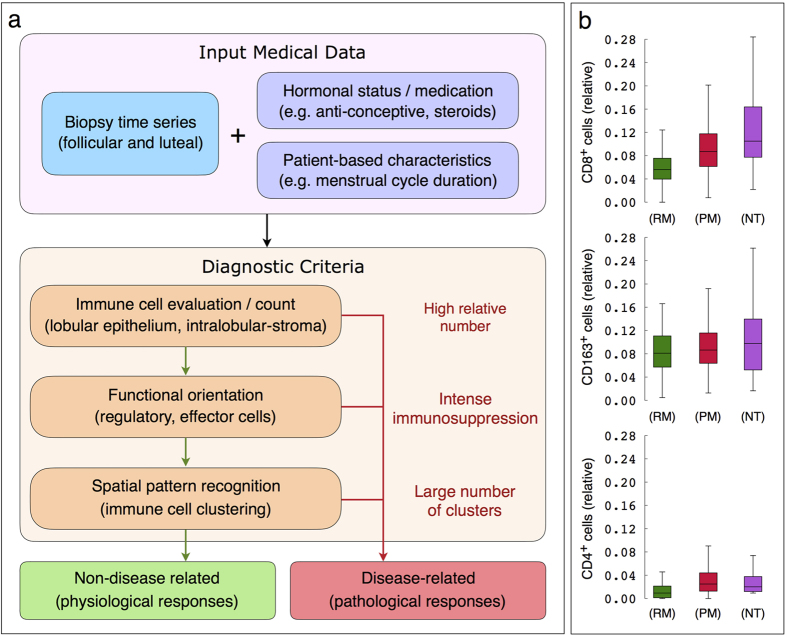
Model-driven diagnostic criteria. (**a**) Immunological evaluation in breast lobular tissue with respect to immune cell density, functional orientation and spatial distribution. (**b**) Quantification of immune cells in breast lobular tissue from women who underwent reduction mammoplasty (RM) due to orthopedic or cosmetic reasons, and prophylactic mastectomy (PM) due to *BRCA1*/*2* mutations. Amount of immune cells in normal lobular tissue adjacent (more than 1 mm) to breast cancer/neoplastic tissue (NT) is also quantified. From top to bottom, the relative number of CD8^+^, CD163^+^ and CD4^+^ cells with respect to epithelial cells. Each box is drawn around the region between the first and third quartiles of the data points, with a horizontal line at the median value and whiskers extend for a range equal to 1.5 times the interquartile range.

As stated above, we found out that the density, functional orientation and spatial distribution of immune cells provide valuable information for the prediction of potential pathological scenarios. These immune-related features can be, for instance, extrapolated from digital whole-slide images of immunohistochemical epithelial and immune cell markers by image analysis processing. Immune infiltrate density, within lobular structures, is considered as the *traditional* diagnostic criterion, since high concentration of lymphocytes suggests pathological conditions. However, model simulations suggest that just an *increased* infiltrate density is not enough to distinguish a severe, but periodically resolving from a chronic potentially persisting, and a chronic (persistent) inflammatory response. The next feature of interest would be the functional orientation of immune cells, that could be captured for example in the form of differences in the balance between effector and regulatory cell populations. Even though highly immunosuppressive microenvironment in breast lobular areas, hypothetically represented by a greater difference, should suggest chronic inflammations, intermediate values are probably not conclusive. Further in-depth studies including more detailed sub-typing of immune cells are required to clarify those questions. We realize that our model is working under the greatly simplified assumption that all detected regulatory cells are equally promoting a suppressing T-effector cell function. While this is a reasonable simplification for physiological situations, we will need to diversify the range of regulatory *agents* for applications modeling pathological conditions such as tumors or their precursor lesions. In addition, we propose to characterize the spatial distribution of immune cells in terms of lymphocyte clustering formation. According to our *in silico* results, in the case of an unusually high amount of immune infiltrates, intense immunosuppression and a large number of clusters can safely suggest that lobular inflammation is pathological, and further clinical actions need to be taken. Figure 12a summarizes the model-driven proposal for immunological context evaluation in breast lobular tissue.

As a proof of principle, we compare small cohorts of breast tissue samples from women who underwent (i) reduction mammoplasty (RM) and (ii) prophylactic mastectomy (PM) due to an increased risk for hereditary breast cancer (*BRCA1/2* mutations), as well as (iii) normal lobular tissue adjacent to neoplastic tissue (NT) from women with clinically manifest breast cancer. In this order, the afore-mentioned categories typically exhibit increased rates of epithelial damaged, e.g. potentially as a result of increasingly accumulated germ line aberrations. This additional experimental data supports the model predictions of increased amount of infiltrating immune cells, both effector and regulatory cells (Fig. 12b), as well as more immune infiltrates localized in contact and close to lobular epithelium (Supplemental Fig. S12), for increasing epithelial damage rates. Additionally, we observe that epithelial damage induces higher variations in the amount of immune infiltrates as suggested by the model simulations. An improved characterization of immune infiltrate spatial patterns in larger cohorts of normal and neoplastic tissue samples, integrating relevant personalized clinical data, will be the subject of future work.

In summary, the model analysis provided a better understanding of the role of inflammation in breast lobular epithelium, emphasized the prognostic value of the menstrual cycle length and hormone levels, and linked spatial distributions of immune infiltrates to potentially pathological scenarios. Although the biological processes involved and the immunological landscape are expected to be much more complicated than the current model description, we believe that our findings and the proposed modeling framework are important steps forward towards extended, more comprehensive criteria for breast tissue biopsy evaluation, specifically supporting the development of novel prognostic markers, and companion biomarkers guiding for immunomodulatory therapeutic interventions.

## Additional Information

**How to cite this article**: Alfonso, J. C. L. *et al*. In-silico insights on the prognostic potential of immune cell infiltration patterns in the breast lobular epithelium. *Sci. Rep.* **6**, 33322; doi: 10.1038/srep33322 (2016).

## Supplementary Material

Supplementary Information

## Figures and Tables

**Figure 1:**
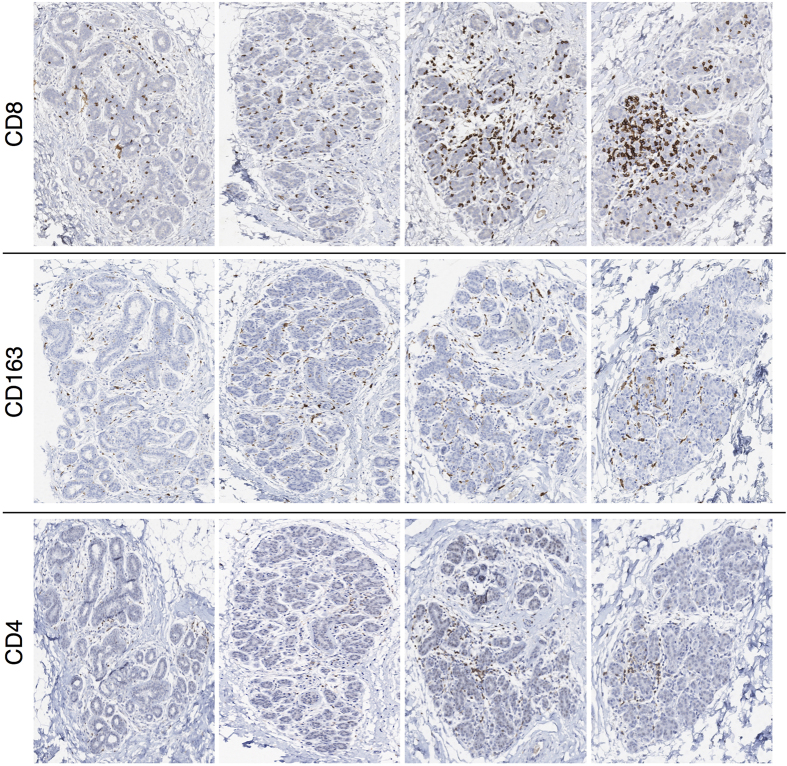
The range of infiltration patterns within breast tissue from one healthy woman. From top to bottom, immunohistochemical stainings for CD8, CD163 and CD4. From left to right, a representative spectrum of immune cell infiltration in different lobular structures for almost consecutive tissue sections by columns. The breast tissue samples were obtained from reduction mammoplasty in a 25-year-old premenopausal woman without clinical abnormality nor familial history of breast cancer, who underwent the surgical procedure during the luteal phase of her regular menstrual cycle, reported to be 30 days long.

**Figure 2:**
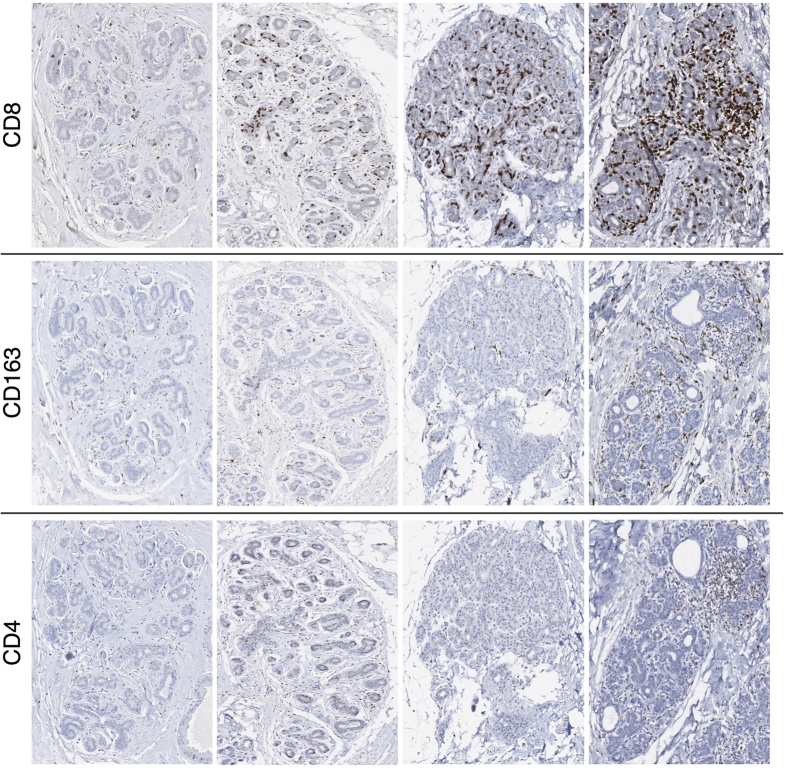
The range of infiltration patterns across breast tissue samples from several healthy women. From top to bottom, immunohistochemical stainings for CD8, CD163 and CD4. From left to right, a representative spectrum of immune cell infiltration in different lobular structures for almost consecutive tissue sections by columns. The breast tissue samples show representative variations as observed in premenopausal women without clinical abnormality nor familial history of breast cancer, with ages ranging from 20 to 33 years (median age 24.5 years), who underwent the surgical procedures during the luteal phase of their regular menstrual cycles, reported to be from 25 to 37 days long (median 28 days).

## References

[b1] Andres A.-C. & Strange R. Apoptosis in the estrous and menstrual cycles. Journal of Mammary Gland Biology and Neoplasia 4, 221–228 (1999). 10426401 10.1023/a:1018737510695

[b2] Ferguson D. & Anderson T. Morphological evaluation of cell turnover in relation to the menstrual cycle in the “resting” human breast. British Journal of Cancer 44, 177 (1981). 7272186 10.1038/bjc.1981.168 PMC2010743

[b3] Anderson T., Ferguson D. & Raab G. Cell turnover in the “resting” human breast: influence of parity, contraceptive pill, age and laterality. British Journal of Cancer 46, 376 (1982). 7126427 10.1038/bjc.1982.213 PMC2011125

[b4] Going J., Anderson T., Battersby S. & MacIntyre C. Proliferative and secretory activity in human breast during natural and artificial menstrual cycles. The American Journal of Pathology 130, 193 (1988). 3337211 PMC1880536

[b5] Potten C. S. . The effect of age and menstrual cycle upon proliferative activity of the normal human breast. British Journal of Cancer 58, 163 (1988). 3166907 10.1038/bjc.1988.185 PMC2246757

[b6] Ramakrishnan R., Khan S. A. & Badve S. Morphological changes in breast tissue with menstrual cycle. Modern Pathology 15, 1348–1356 (2002). 12481017 10.1097/01.MP.0000039566.20817.46

[b7] Navarrete M. . Assessment of the proliferative, apoptotic and cellular renovation indices of the human mammary epithelium during the follicular and luteal phases of the menstrual cycle. Breast Cancer Research 7, R306–13 (2005). 10.1186/bcr994 PMC1143573 15987425

[b8] Russo J. & Russo I. H. Histological evaluation of the normal breast. In Techniques and Methodological Approaches in Breast Cancer Research 45–73 (Springer, 2014).

[b9] Merlino G. Regulatory imbalances in cell proliferation and cell death during oncogenesis in transgenic mice. Seminars in Cancer Biology 5, 13–20 (1994). 8186384

[b10] Liu S., Edgerton S. M., Moore D. H. & Thor A. D. Measures of cell turnover (proliferation and apoptosis) and their association with survival in breast cancer. Clinical Cancer Research 7, 1716–1723 (2001). 11410511

[b11] Labi V. & Erlacher M. How cell death shapes cancer. Cell Death & Disease 6, e1675 (2015). 25741600 10.1038/cddis.2015.20 PMC4385913

[b12] Gorgoulis V. G. . Activation of the dna damage checkpoint and genomic instability in human precancerous lesions. Nature 434, 907–913 (2005). 15829965 10.1038/nature03485

[b13] Caldon C. Estrogen signaling and the dna damage response in hormone dependent breast cancers. Front Oncology 4, 1–9 (2014). 10.3389/fonc.2014.00106 PMC4030134 24860786

[b14] Nandi S., Guzman R. C. & Yang J. Hormones and mammary carcinogenesis in mice, rats, and humans: a unifying hypothesis. Proceedings of the National Academy of Sciences 92, 3650–3657 (1995). 10.1073/pnas.92.9.3650 PMC42019 7731959

[b15] Martin L., Coffey M., Lawler M., Hollywood D. & Marignol L. Dna mismatch repair and the transition to hormone independence in breast and prostate cancer. Cancer Letters 291, 142–149 (2010). 19896265 10.1016/j.canlet.2009.10.007

[b16] Rose-Hellekant T. A., Wentworth K. M., Nikolai S., Kundel D. W. & Sandgren E. P. Mammary carcinogenesis is preceded by altered epithelial cell turnover in transforming growth factor-α and c-myc transgenic mice. The American Journal of Pathology 169, 1821–1832 (2006). 17071603 10.2353/ajpath.2006.050675 PMC1780202

[b17] Coussens L. M. & Pollard J. W. Leukocytes in mammary development and cancer. Cold Spring Harbor Perspectives in Biology 3, a003285 (2011). 21123394 10.1101/cshperspect.a003285 PMC3039933

[b18] Polyak K. Breast cancer: origins and evolution. The Journal of Clinical Investigation 117, 3155–3163 (2007). 17975657 10.1172/JCI33295 PMC2045618

[b19] Jackson S. P. & Bartek J. The dna-damage response in human biology and disease. Nature 461, 1071–1078 (2009). 19847258 10.1038/nature08467 PMC2906700

[b20] Roy R., Chun J. & Powell S. N. Brca1 and brca2: different roles in a common pathway of genome protection. Nature Reviews Cancer 12, 68–78 (2012). 22193408 10.1038/nrc3181 PMC4972490

[b21] Petrucelli N., Daly M. B. & Feldman G. L. Hereditary breast and ovarian cancer due to mutations in brca1 and brca2. Genetics in Medicine 12, 245–259 (2010). 20216074 10.1097/GIM.0b013e3181d38f2f

[b22] Narod S. Modifiers of risk of hereditary breast cancer. Oncogene 25, 5832–5836 (2006). 16998497 10.1038/sj.onc.1209870

[b23] Smith T. R., Miller M. S., Lohman K. K., Case L. D. & Hu J. J. Dna damage and breast cancer risk. Carcinogenesis 24, 883–889 (2003). 12771032 10.1093/carcin/bgg037

[b24] Hanahan D. & Weinberg R. A. The hallmarks of cancer. Cell 100, 57–70 (2000). 10647931 10.1016/s0092-8674(00)81683-9

[b25] Greenman C. . Patterns of somatic mutation in human cancer genomes. Nature 446, 153–158 (2007). 17344846 10.1038/nature05610 PMC2712719

[b26] Hanahan D. & Weinberg R. A. Hallmarks of cancer: the next generation. Cell 144, 646–674 (2011). 21376230 10.1016/j.cell.2011.02.013

[b27] Degnim A. C. . Immune cell quantitation in normal breast tissue lobules with and without lobulitis. Breast Cancer Research and Treatment 144, 539–549 (2014). 24596048 10.1007/s10549-014-2896-8 PMC3962744

[b28] Smyth M. J., Dunn G. P. & Schreiber R. D. Cancer immunosurveillance and immunoediting: the roles of immunity in suppressing tumor development and shaping tumor immunogenicity. Advances in Immunology 90, 1–50 (2006). 16730260 10.1016/S0065-2776(06)90001-7

[b29] Shankaran V. . Ifnγ and lymphocytes prevent primary tumour development and shape tumour immunogenicity. Nature 410, 1107–1111 (2001). 11323675 10.1038/35074122

[b30] Atabai K., Sheppard D. & Werb Z. Roles of the innate immune system in mammary gland remodeling during involution. Journal of mammary gland biology and neoplasia 12, 37–45 (2007). 17286210 10.1007/s10911-007-9036-6 PMC2574498

[b31] Chetty R. & Butler A. Lymphocytic mastopathy associated with infiltrating lobular breast carcinoma. Journal of Clinical Pathology 46, 376–377 (1993). 8496398 10.1136/jcp.46.4.376 PMC501226

[b32] Lee A., Happerfield L., Millis R. & Bobrow L. Inflammatory infiltrate in invasive lobular and ductal carcinoma of the breast. British Journal of Cancer 74, 796 (1996). 8795584 10.1038/bjc.1996.438 PMC2074701

[b33] Hermsen B. B. . Lobulitis is a frequent finding in prophylactically removed breast tissue from women at hereditary high risk of breast cancer. The Journal of Pathology 206, 220–223 (2005). 15880615 10.1002/path.1774

[b34] Douglas-Jones A. Lymphocytic lobulitis in breast core biopsy: a peritumoral phenomenon. Histopathology 48, 209–212 (2006). 16405676 10.1111/j.1365-2559.2005.02212.x

[b35] Gulbahce H. E., Vanderwerf S., Blair C. & Sweeney C. Lobulitis in nonneoplastic breast tissue from breast cancer patients: association with phenotypes that are common in hereditary breast cancer. Human Pathology 45, 78–84 (2014). 24157064 10.1016/j.humpath.2013.08.008

[b36] DeNardo D. G. & Coussens L. M. Balancing immune response: crosstalk between adaptive and innate immune cells during breast cancer progression. Breast Cancer Research 9, 212 (2007). 17705880 10.1186/bcr1746 PMC2206719

[b37] Pages F. . Immune infiltration in human tumors: a prognostic factor that should not be ignored. Oncogene 29, 1093–1102 (2010). 19946335 10.1038/onc.2009.416

[b38] Pollard J. W. Tumour-educated macrophages promote tumour progression and metastasis. Nature Reviews Cancer 4, 71–78 (2004). 14708027 10.1038/nrc1256

[b39] Balkwill F. & Mantovani A. Inflammation and cancer: back to virchow? The lancet 357, 539–545 (2001). 10.1016/S0140-6736(00)04046-0 11229684

[b40] Grivennikov S. I., Greten F. R. & Karin M. Immunity, inflammation, and cancer. Cell 140, 883–899 (2010). 20303878 10.1016/j.cell.2010.01.025 PMC2866629

[b41] Coussens L. M. & Werb Z. Inflammation and cancer. Nature 420, 860–867 (2002). 12490959 10.1038/nature01322 PMC2803035

[b42] Balkwill F., Charles K. A. & Mantovani A. Smoldering and polarized inflammation in the initiation and promotion of malignant disease. Cancer Cell 7, 211–217 (2005). 15766659 10.1016/j.ccr.2005.02.013

[b43] Feuerhake F., Sigg W., Höfter E., Unterberger P. & Welsch U. Cell proliferation, apoptosis, and expression of bcl-2 and bax in non-lactating human breast epithelium in relation to the menstrual cycle and reproductive history. Breast Cancer Research and Treatment 77, 37–48 (2003). 12602903 10.1023/a:1021119830269

[b44] Brieu N., Pauly O., Zimmermann J., Binnig G. & Schmidt G. Slide specific models for segmentation of differently stained digital histopathology whole slide images. Proc. SPIE 9784, Medical Imaging: Image Processing, vol. 9784, 978410 (2016).

[b45] Gudjonsson T., Adriance M. C., Sternlicht M. D., Petersen O. W. & Bissell M. J. Myoepithelial cells: their origin and function in breast morphogenesis and neoplasia. Journal of Mammary Gland biology and Neoplasia 10, 261–272 (2005). 16807805 10.1007/s10911-005-9586-4 PMC2798159

[b46] Russo J., Ao X., Grill C. & Russo I. Pattern of distribution of cells positive for estrogen receptor α and progesterone receptor in relation to proliferating cells in the mammary gland. Breast Cancer Research and Treatment 53, 217–227 (1999). 10369068 10.1023/a:1006186719322

[b47] Gjorevski N. & Nelson C. M. Integrated morphodynamic signalling of the mammary gland. Nature Reviews Molecular Cell Biology 12, 581–593 (2011). 21829222 10.1038/nrm3168

[b48] Brisken C. Progesterone signalling in breast cancer: a neglected hormone coming into the limelight. Nature Reviews Cancer 13, 385–396 (2013). 23702927 10.1038/nrc3518

[b49] Jones C. . Expression profiling of purified normal human luminal and myoepithelial breast cells identification of novel prognostic markers for breast cancer. Cancer Research 64, 3037–3045 (2004). 15126339 10.1158/0008-5472.can-03-2028

[b50] Salgado R. . The evaluation of tumor-infiltrating lymphocytes (tils) in breast cancer: recommendations by an international tils working group 2014. Annals of Oncology 26, 259–271 (2015). 25214542 10.1093/annonc/mdu450 PMC6267863

[b51] Andersen M. H., Schrama D., thor Straten P. & Becker J. C. Cytotoxic t cells. Journal of Investigative Dermatology 126, 32–41 (2006). 16417215 10.1038/sj.jid.5700001

[b52] Wiedemann A., Depoil D., Faroudi M. & Valitutti S. Cytotoxic t lymphocytes kill multiple targets simultaneously via spatiotemporal uncoupling of lytic and stimulatory synapses. Proceedings of the National Academy of Sciences 103, 10985–10990 (2006). 10.1073/pnas.0600651103 PMC1544161 16832064

[b53] Janeway C. A., Travers P., Walport M., Shlomchik M. J. . Immunobiology: the immune system in health and disease vol. 2 (Garland: New York, , 2001).

[b54] Trapani J. A. & Smyth M. J. Functional significance of the perforin/granzyme cell death pathway. Nature Reviews Immunology 2, 735–747 (2002). 10.1038/nri911 12360212

[b55] Medrek C., Pontén F., Jirström K. & Leandersson K. The presence of tumor associated macrophages in tumor stroma as a prognostic marker for breast cancer patients. BMC Cancer 12, 1 (2012). 22824040 10.1186/1471-2407-12-306 PMC3414782

[b56] Murdoch C., Muthana M., Coffelt S. B. & Lewis C. E. The role of myeloid cells in the promotion of tumour angiogenesis. Nature Reviews Cancer 8, 618–631 (2008). 18633355 10.1038/nrc2444

[b57] DeNardo D. G. . Leukocyte complexity predicts breast cancer survival and functionally regulates response to chemotherapy. Cancer Discovery 1, 54–67 (2011). 22039576 10.1158/2159-8274.CD-10-0028 PMC3203524

[b58] Zou W. Regulatory t cells, tumour immunity and immunotherapy. Nature Reviews Immunology 6, 295–307 (2006). 10.1038/nri1806 16557261

[b59] Ostrand-Rosenberg S. Immune surveillance: a balance between protumor and antitumor immunity. Current Opinion in Genetics & Development 18, 11–18 (2008). 18308558 10.1016/j.gde.2007.12.007 PMC2699403

[b60] Elliott M. R. & Ravichandran K. S. Clearance of apoptotic cells: implications in health and disease. The Journal of cell biology 189, 1059–1070 (2010). 20584912 10.1083/jcb.201004096 PMC2894449

[b61] Cullen S. P. . Fas/cd95-induced chemokines can serve as “find-me” signals for apoptotic cells. Molecular Cell 49, 1034–1048 (2013). 23434371 10.1016/j.molcel.2013.01.025

[b62] Chao M. P., Majeti R. & Weissman I. L. Programmed cell removal: a new obstacle in the road to developing cancer. Nature Reviews Cancer 12, 58–67 (2012). 22158022 10.1038/nrc3171

[b63] Green D. R. & Levine B. To be or not to be? how selective autophagy and cell death govern cell fate. Cell 157, 65–75 (2014). 24679527 10.1016/j.cell.2014.02.049 PMC4020175

[b64] Rodier F. . Persistent dna damage signalling triggers senescence-associated inflammatory cytokine secretion. Nature cell biology 11, 973–979 (2009). 19597488 10.1038/ncb1909 PMC2743561

[b65] Malaquin N., Carrier-Leclerc A., Dessureault M. & Rodier F. Ddr-mediated crosstalk between dna-damaged cells and their microenvironment. Frontiers in genetics 6 (2015). 10.3389/fgene.2015.00094 PMC4357297 25815006

[b66] Kidane D. . Interplay between dna repair and inflammation, and the link to cancer. Critical reviews in biochemistry and molecular biology 49, 116–139 (2014). 24410153 10.3109/10409238.2013.875514 PMC4300235

[b67] Chatzinikolaou G., Karakasilioti I. & Garinis G. A. Dna damage and innate immunity: links and trade-offs. Trends in immunology 35, 429–435 (2014). 25023467 10.1016/j.it.2014.06.003

[b68] Thelen M. & Stein J. V. How chemokines invite leukocytes to dance. Nature Immunology 9, 953–959 (2008). 18711432 10.1038/ni.f.207

[b69] Moser B. & Loetscher P. Lymphocyte traffic control by chemokines. Nature Immunology 2, 123–128 (2001). 11175804 10.1038/84219

[b70] Esche C., Stellato C. & Beck L. A. Chemokines: key players in innate and adaptive immunity. Journal of Investigative Dermatology 125, 615–628 (2005). 16185259 10.1111/j.0022-202X.2005.23841.x

[b71] Baggiolini M. Chemokines and leukocyte traffic. Nature 392, 565–568 (1998). 9560152 10.1038/33340

[b72] Stampfer M. R. & Bartley J. C. Induction of transformation and continuous cell lines from normal human mammary epithelial cells after exposure to benzo [a] pyrene. Proceedings of the National Academy of Sciences 82, 2394–2398 (1985). 10.1073/pnas.82.8.2394 PMC397564 3857588

[b73] Band V. & Sager R. Distinctive traits of normal and tumor-derived human mammary epithelial cells expressed in a medium that supports long-term growth of both cell types. Proceedings of the National Academy of Sciences 86, 1249–1253 (1989). 10.1073/pnas.86.4.1249 PMC286665 2919173

[b74] Mukhopadhyay R. . Promotion of variant human mammary epithelial cell outgrowth by ionizing radiation: an agent-based model supported by in vitro studies. Breast Cancer Research 12, R11 (2010). 20146798 10.1186/bcr2477 PMC2880432

[b75] Hansen J.-P. & McDonald I. R. Theory of simple liquids (Elsevier, 1990).

[b76] Degnim A. C. . Histologic findings in normal breast tissues: comparison to reduction mammaplasty and benign breast disease tissues. Breast cancer research and treatment 133, 169–177 (2012). 21881938 10.1007/s10549-011-1746-1 PMC3242875

[b77] Shin D. S. & Ribas A. The evolution of checkpoint blockade as a cancer therapy: what’s here, what’s next? Current Opinion in Immunology 33, 23–35 (2015). 25621841 10.1016/j.coi.2015.01.006

[b78] Rakha E. A. . Prognostic significance of nottingham histologic grade in invasive breast carcinoma. Journal of Clinical Oncology 26, 3153–3158 (2008). 18490649 10.1200/JCO.2007.15.5986

[b79] Savas P. . Clinical relevance of host immunity in breast cancer: from tils to the clinic. Nature Reviews Clinical Oncology (2015). 10.1038/nrclinonc.2015.215 26667975

[b80] Medh R. D. & Thompson E. B. Hormonal regulation of physiological cell turnover and apoptosis. Cell and Tissue Research 301, 101–124 (2000). 10928284 10.1007/s004419900159 PMC2763512

