# Supplementary material for: In-silico insights on the prognostic potential of immune cell infiltration patterns in the breast lobular epithelium
Source: Sci Rep. 2016 Sep 23;6:33322. doi: 10.1038/srep33322 (PMC5034260; doi:10.1038/srep33322)
Supplement: Supplementary Information [file srep33322-s1.pdf]

In-silico insights on the prognostic potential of immune cell infiltration patterns in the breast lobular epithelium

J. C. L. Alfonso[1,2], N. S. Schaadt[3], R. Schönmeyer[4], N. Brieu[4], G. Forestier[5,6], C. Wemmert[5], F. Feuerhake[3,7], and H. Hatzikirou[1,2,*]

[1]Center for Information Services and High Performance Computing, Technische Universität Dresden, 01062 Dresden, Germany.
[2]Braunschweig Integrated Centre of Systems Biology, Helmholtz Center for Infectious Research, 38124 Braunschweig, Germany.
[3]Institute for Pathology, Hannover Medical School, 30625 Hannover, Germany.
[4]Definiens AG, 80636 Munich, Germany.
[5]Engineering Science, Computer Science and Imaging Laboratory, Université de Strasbourg, 67400 Strasbourg, France.
[6]Modelling, Intelligence, Process and Systems, Université de Haute Alsace, 68093 Mulhouse, France.
[7]Institute of Neuropathology, University Clinic Freiburg, 79117 Freiburg, Germany.
[*]haralampos.hatzikirou@helmholtz-hzi.de

SUPPORTING INFORMATION

We develop a multiscale agent-based model of epithelial and immune cell interactions based on cyclic changes of cell turnover in breast lobular tissue. This document contains details about model formulation, parametrization and implementation, as well as additional simulation results.

Cell types and states

The model accounts for myoepithelial and luminal cells that comprise the breast lobular epithelium, as well as CD163$^+$ macrophages, CD4$^+$ cells and CD8$^+$ T-lymphocytes as part of the immune system. We assume that epithelial cells may be either in a normal, damaged or dying state. Moreover, damaged and dying epithelial cells release two different chemokines (or chemotactic cytokines) inducing immune responses. Immune cells become activated by chemokines from damaged epithelial cells. On the other hand, activated immune cells become inactive in the absence of such chemoattractant signals or due to the suppressing action of regulatory cells in the case of effectors. In a simplified approach, we assign the following functions to different immune cell subtypes, realizing that this can only be an approximation to the true complexity of immune regulation: while *effector* CD8$^+$ cells are the only responsible for killing (*target*) damaged epithelial cells, the function of *regulatory* CD4$^+$ and CD163$^+$ cells is a general suppression of effector-dependent responses by inducing inactivation. Fig. S1 shows a schematic representation of cell types, states and immune cell interactions in the agent-based model.

Breast lobular epithelium geometry and simulation domain

The simulation domain represents cross-sections of terminal ductal lobular units (TDLUs) composed by several terminal ductules/acini, each with an inner layer of luminal cells surrounded by an exterior layer of myoepithelial cells (Fig. S2a,b). A cross-section of TDLUs is defined by a polygon delimiting the intra- and interlobular stroma (Fig. S2b). In the mammary gland, as well as in other epithelial structures, cells are polarized into apical and basal cell compartments, connected by physical bonds such as tight junctions. The basal membrane keeps epithelial cells neatly organized in their functional structure, clearly separated from the underlying connective and fat tissue.[1,2] Accordingly, luminal and myoepithelial cells are assumed firmly placed within the breast lobular epithelium.

Each terminal ductule is defined by three equally centered ellipses (Fig. S2c). While the interior ellipse delimits the lobular lumina, the intermediate and exterior ellipses define the luminal and myoepithelial cell compartments, respectively. The major and minor axes of the ellipses, as well as the position and orientation, are arbitrary selected to simulate cross-sections of TDLUs as in breast tissue samples. The width of the epithelial cell compartments is considered of about one cell diameter as experimentally observed (Fig. S2a,b).

For computer simulations, a two-dimensional (2D) square lattice is first defined for epithelial cells, referred to as the epithelial-lattice. Then, an additional 2D lattice is created for immune cells, i.e. the immune-lattice, by placing exactly one

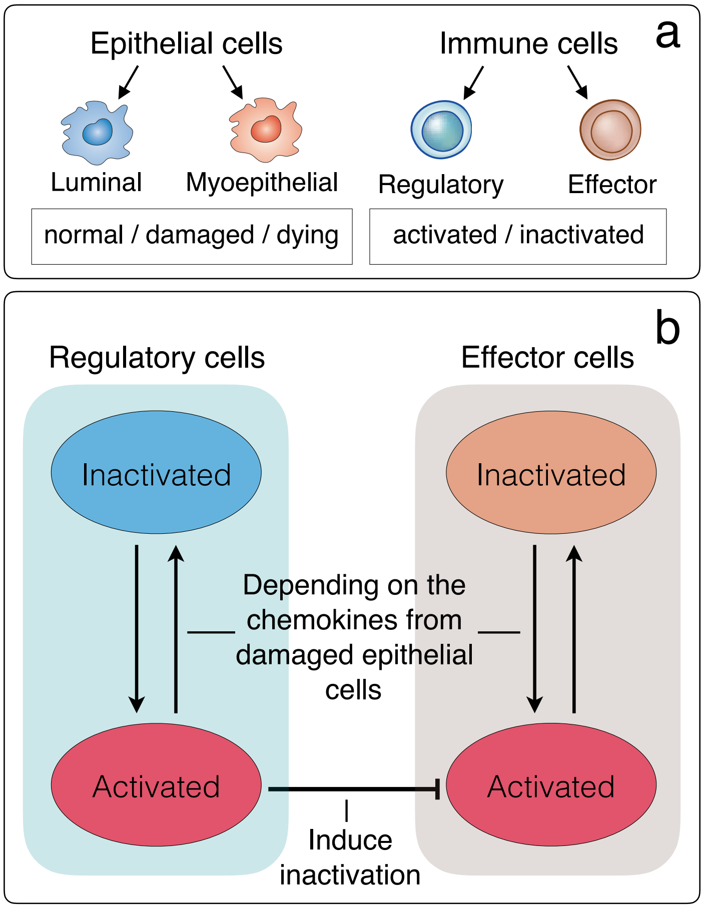

Figure S1. **Cell types, states and immune cell interactions**. **(a)** Epithelial and immune cell types and states in the agent-based model. **(b)** Immune cell activation / inactivation transitions depending on chemokines from damaged epithelial cells, as well as inactivation of effectors by the suppressing action of regulatory cells.

node at the center of each square in the epithelial-lattice (Fig. S2d). The position of each node in both lattices is slightly and randomly modified avoiding symmetry artifacts. A Moore neighbourhood is considered for nodes in the same lattice, i.e. the eight orthogonally and diagonally adjacent nodes. Moreover, each node has four neighbours in the opposing lattice defined by the corners of the square in which they are located. Fig. S2d shows the neighbours of a node in the epithelial-lattice, where a similar neighbourhood is defined for nodes in the immune-lattice. This particularity in the construction of the simulation domain avoids contact inhibition between immune and epithelial cells, and allows to simulate the motility of lymphocytes through the breast lobular epithelium. We consider that each node can host only one cell at any simulation time. Furthermore, immune and epithelial cells only reside in their respective lattices, and therefore jump between lattices is not allowed.

The lattice constant a, defined by the distance between nearest and orthogonal neighbor nodes in a same lattice, is chosen such that the average area of a simulation cell $S[A] = a^2$ corresponds to that of a biological cell $A = (\pi/4)l^2$. Then, considering an average cell diameter l equal to 25 μm for mammary epithelial cells and lymphocytes,[3,4] we have that $a = (\pi/4)^{1/2} l \approx 20$ μm. The simulation domain is divided into 150×200 nodes in the epithelial-lattice corresponding to an area of about 10 mm^2, and accounts for four different cross-sections of TDLUs (Fig. 3d,e). Actually, the selected modeling framework permits simulations to be scaled up to square centimetres for several cross-sections of TDLUs, although at the expense of higher computational resources.

Spatio-temporal dynamic of chemokine concentrations

Chemokines are small cell-secreted protein molecules involved in mechanisms like maturation, traffic, homing and retention of immune cells including monocytes/macrophages, regulatory and effector T-cells, which express chemokine receptors mediating

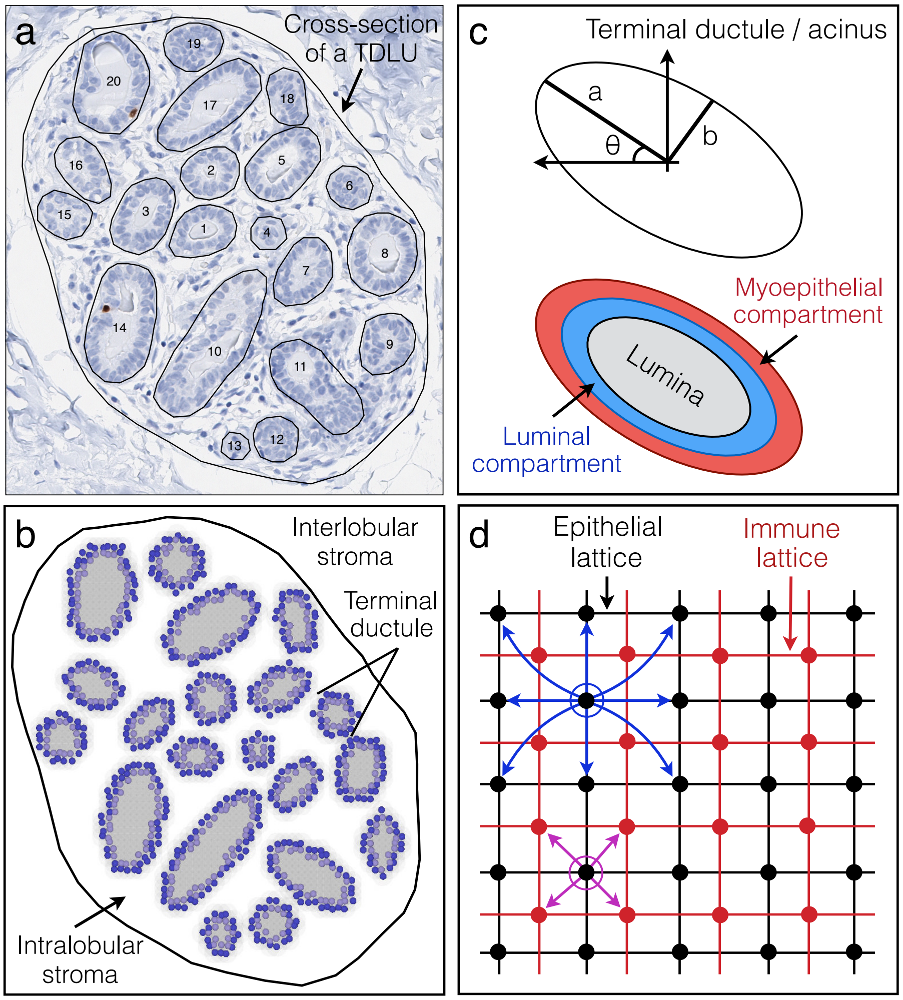

Figure S2. Model simulation details. (A) Cross-section of a terminal ductal lobular unit (TDLU) and (B) the corresponding simplified simulation domain. (C) Representation of a terminal ductule, as well as the myoepithelial and luminal cell compartments which delimit the lobular lumina. (D) Schematic representation of the interconnected lattices in the simulation domain. In blue the eight neighbours (same lattice) of a node in the epithelial-lattice. In purple the four neighbours (opposing lattice) in the immune-lattice.

their cellular effects.[5–7] Chemokine gradients are seen as the stimuli that largely control leukocyte migration to sites of inflammation and target cell recognition by a process called chemotaxis.[5–8] In fact, chemokines favor the induction of an efficient immune response, and are now recognized to be involved in a wide variety of leukocyte functions during inflammation and immunity.[9] Given that such chemokine molecules are very small compared with the size of cells, we simulate them as continuous and use a partial differential equation to model concentration changes in space and time. Denoting the concentration of chemokines as c, either released by damaged or dying epithelial cells, we have that

$$\frac{\partial c}{\partial t} = D_c \nabla^2 c + rc(1-c)\delta(x) - dc, \tag{1}$$

where D_c is the diffusion coefficient, r is the secretion rate per cell, d is the natural decay rate and $\delta(x)$ is a characteristic function equal to 1 if there is a damaged or dying epithelial cell in x, and zero in other case. The model parameters D_c, r and d are positive constants.

Chemokines diffuse several orders of magnitude faster than cells.[10] Estimates of D_c have been reported to vary from 10^{-9} to 10^{-6} cm^2/s.[10–14] The natural decay rate of chemokines d has been reported to vary from 10^{-4} to 10^{-3} s^{-1},[14] which is in

agreement with estimates of the half-life of various chemokines varying from 25 to 90 minutes.[15,16] The secretion rate of chemokines from a signaling cell falls into the range of 20 to 3000 molecules per cell and minute.[10] From these ranges of parameter values, we take $D_c = 3.0 \times 10^2$ μm^2 h^{-1}, $d = 3.0 \times 10^{-1}$ h^{-1} and $r = 5.0 \times 10^4$ molecules cell^{-1} h^{-1}.

We solve Equation (1) in a simulation domain given by the nodes in both the immune- and epithelial-lattice. As initial condition for signaling epithelial cells we consider $c_0 = \bar{c} = 10^{-5}$ molecules cell^{-1} h^{-1}, as well as no-flux conditions $\partial c/\partial x = 0$ in the boundary $\partial\Omega$ of the rectangular simulation domain Ω. For the discretization of the diffusion term in Equation (1) we used the central explicit finite difference scheme. More precisely, we implemented the forward-difference and second-order central-difference numerical approximations for $\partial c/\partial t$ and $\nabla^2 c$, respectively.[17] Stable and physically meaningful solutions are obtained as long as the two-dimensional Courant-Friedrichs-Lewy (CFL) condition $\Delta t \leq h^2/4D_c$ is fulfilled,[18] where Δt is the required discrete time-step and $h = 20$ μm. Since the simulation time-step is one hour and $D_c = 3.0 \times 10^2$ μm^2 h^{-1}, the chemokine concentrations are updated three times per time-step (every 20 minutes) to satisfy the CFL condition.

Cell processes

The cell processes considered in the agent-based model are proliferation of epithelial cells, immune cell motility, death of damaged epithelial cells by effectors, inactivation of effectors by regulatory cells, programmed cell death (apoptosis) and lysis (removal of cellular debris) (Fig. 4a). We provide here a detailed description of these cellular processes, as well as we explain the choice of the corresponding parameter values.

- **Motility**. The movement of immune cells is only limited to the lumina of terminal ductules. An immune cell placed in a node with no free neighbor nodes in the immune-lattice temporarily loses its ability to move due to contact inhibition. This quiescent state may be abandoned as soon as one of the neighbor nodes becomes free. It is known that epithelial cells exhibit a significantly low motility rate compared to immune cells, as they are firmly placed within the breast lobular epithelium.[1,2] Accordingly, we have considered that epithelial cells do not move.

  Inactivated immune cells move randomly to a free neighbor node in the immune-lattice in the absence of chemoattractant signals. Otherwise, immune cells describe a chemotactic movement to a free neighbor node with a certain probability. In fact, activated and inactivated immune cells follow the chemoattractant signal from damaged and dying epithelial cells, respectively. The transition probability for the chemotactic movement of a cell from a node r to a free neighbor node r' is determined by

$$p(r \to r') = \frac{e^{\delta \Delta S}}{\sum\limits_{r \in N(r)} e^{\delta \Delta S}}, \tag{2}$$

  where $N(r)$ is the set of free neighbor nodes of r in the immune-lattice, and $\Delta S = S(r') - S(r)$ with $S(r)$ being the chemokine concentration, either from damaged or dying epithelial cells, in r. The model parameter δ is taken equal to 10 to simulate a non-deterministic biased random walk. A random number is generated to select the free neighbor node for movement based on the distribution set of probabilities given by Equation (2).

  Immune cells are constantly moving inside and between organs, and are considered among the most motile cells in the human body. Estimates of the mean velocity of immune cells in non-lymphoid tissues have been reported at the order of 4 to 10 μm min^{-1}, with a peak velocity as high as 25 μm min^{-1} in the lymph node.[19–21] We consider a velocity of 10 μm min^{-1}, which results in a hopping rate of 0.37 min^{-1} assuming that the waiting time for cell movement follows an exponential probability distribution. Since the simulation time-step is one hour, the movement of immune cells is updated 60 times per time-step (every one minute).

- **Proliferation**. Experimental evidence indicates that in the adult mammary gland, luminal cells are able to give rise to both epithelial cell populations, i.e. luminal and myoepithelial cells, whereas the myoepithelial population only gives rise to myoepithelial cells.[22–25] This in turn suggests that bipotent progenitor cells may reside within the luminal epithelial cell compartment.[25] Accordingly, we assume that luminal cells can also generate daughter myoepithelial cells, but not the other way around.

  Mitosis is only possible for luminal and myoepithelial cells located at a node having at least one free neighbor node in the epithelial compartments. In the case of symmetric cell division, an identical daughter cell is placed on a randomly selected free neighbor node in the same epithelial compartment of the progenitor cell. In addition, luminal cells with at least one

free neighbor node in the myoepithelial compartment can also proliferate, which results in a daughter myoepithelial cell. An epithelial cell placed in a node with no free neighbor nodes temporarily loses its ability to proliferate due to contact inhibition. This quiescent state may be abandoned as soon as one of the neighbor nodes valid for cell proliferation becomes free. For simplicity, we consider that immune cells do not proliferate, and therefore their dynamic is exclusively characterized by cell motility and trafficking.

Estimates of the cell-cycle length of mammary epithelial cells in rats have been reported to vary from 11.65 to 49.63 hours depending on the age, the specific compartment in which each cell type is located and the differentiation cell stages.[26,27] Accordingly, we consider a cell-cycle length, for both myoepithelial and luminal cells, of 26 hours which is in the range reported. This results in an intrinsic proliferation rate $k_{pro} = \ln(2)/26 \approx 0.03$ h^{-1}, which is in line with *in vitro* estimates reported for human mammary epithelial cells.[28–30]

- **Programmed cell death**. Undamaged epithelial cells are subject to apoptosis, i.e. a programmed cell death. When apoptosis occurs, an apoptotic pathway is activated that leads to cell shrinkage, nuclear fragmentation, chromatin condensation and chromosomal DNA fragmentation into apoptotic bodies. We assume that dying epithelial cells produce a chemoattractant signal that induces the recruitment of immune cells.

  Once fixed the intrinsic proliferation rate of epithelial cells k_{pro}, we estimate the intrinsic apoptotic rate k_{apt} to reproduce the reported proliferation index (PI) values with respect to the menstrual cycle.[31] We obtain that $k_{apt} = 0.0021$ h^{-1} provides amounts of proliferating and apoptotic epithelial cells as experimentally quantified.[31]

- **Lysis**. Disposal of cellular debris resulting from apoptosis is carried out by a lysis process that removes dead epithelial cells from the simulation domain.[32] We consider a lysis rate $k_{lys} = 0.035$ h^{-1}, which is less than phagocytosis, i.e. digestion of cellular debris by phagocytes, observed *in vivo*,[33] but within the range reported for *in vitro* cultures between 0.002 h^{-1} for *Hybridoma VO 208* cell line[34] and 0.07 h^{-1} for *Fibrobacter succinogenes*.[35]

- **Immunosuppression**. Activated regulatory cells may permanently inactivate, by direct contact, effector cells at a fixed rate 0.001 h^{-1}. We consider that, together with the update of immune cell movement, inactivation events can occur. Therefore, the immunosuppressive function of activated regulatory cells is also updated 60 times per time-step (every one minute). Moreover, this rate increases proportionally with the number of activated regulatory cells surrounding an activated effector cell. We remark that variations in this parameter value only affect the functional orientation results on a quantitative manner, while the qualitative phenomena are conserved.

Immune cell trafficking

It is well known that one of the main tasks of chemokines is the control of lymphocyte traffic.[37] Leukocytes emigrate from the bloodstream through the venule walls, and follow chemokine gradients by a process called chemotaxis, which allows them to reach sites of inflammation and during normal homing to lymphoid organs.[5–8] The most prominent types of extravasating cells are T-lymphocytes and monocytes.[38]

We assume that the trafficking of immune cells is modulated by chemokines from damaged and dying epithelial cells. More precisely, we first consider that in the absence of chemoattractant signals, the amount of regulatory and effector cells in the breast lobular epithelium remains constant during the menstrual cycle. Under this condition, we simulate the trafficking of immune cells as follows

$$\frac{dN}{dt} = r_A(K - N) - r_D N,$$

where r_A and r_D represent the appearance and disappearance rates of immune cells, and K is the number of nodes in each cross-section of TDLUs or in the interlobular stroma.

Fig. S3 shows that vascular endothelial cells are homogeneously distributed in the breast tissue irrespective of the menstrual cycle phase. In fact, this is in line with the observation that the mammary stroma is made up of highly vascularized loose connective tissue.[39,40] Thus, we assume that immune cells may enter and leave the system through the blood vessels from any node in the simulation domain.

The steady-state approximation of the equation above results in

$$r_A(K - \overline{N}) = r_D\, \overline{N},$$

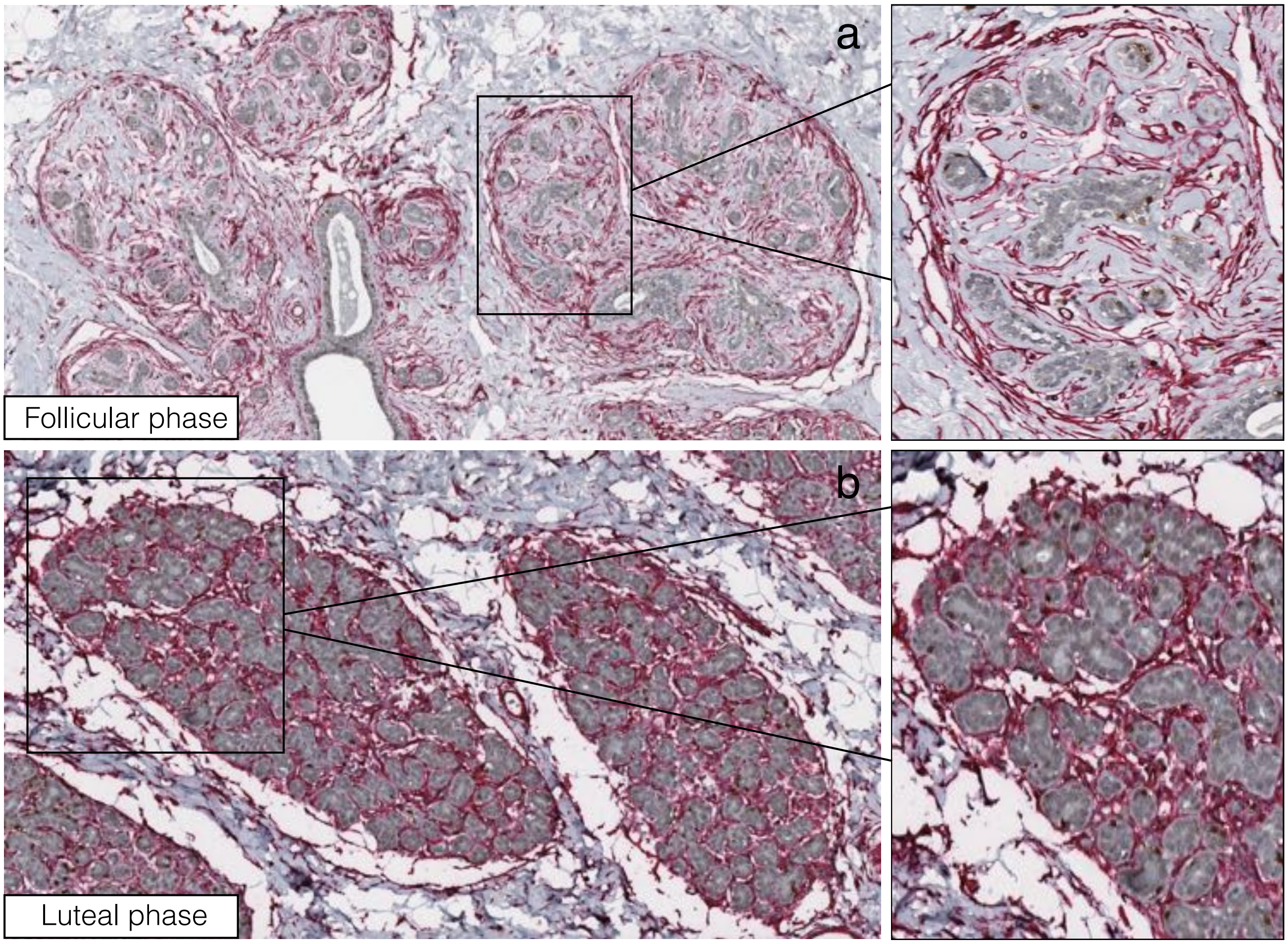

Figure S3. Double immunohistochemical stainings for vascular endothelial cells (CD34; red chromogen) and T-lymphocytes (CD3; brown chromogen) in breast tissue from different healthy women. **(a)** Follicular and **(b)** luteal phases of the menstrual cycle at the time of the biopsies. The breast tissue samples correspond to premenopausal women (27- and 28-year-old) without clinical abnormality nor familial history of breast cancer, and with a menstrual cycle length of 28 days.

where $\overline{N}$ represents the homeostatic number of immune cells in each cross-section of TDLUs or in the interlobular stroma. It follows from this assumption that

$$r_A = \frac{r_D\,\overline{N}}{(K-\overline{N})}.$$

Based on our experimental data, we found that the number of regulatory cells does not vary significantly during the menstrual cycle compared to the amount of effector cells (Fig. S6). Thus, we assume that their trafficking is not affected by the chemoattractant signal from dying epithelial cells. The experimental mean relative number of regulatory cells per 1000 epithelial cells is approximately 0.055. Thus, $\overline{N}_{\text{reg}}$ is selected such that the amount of regulatory cells on each cross-section of TDLUs satisfies this estimate. On the other hand, the amount of effector cells significantly change during the menstrual cycle (Fig. S6). We selected $\overline{N}_{\text{eff}}$ on each cross-section of TDLUs to satisfy a ratio of 0.025 effectors per 1000 of epithelial cells as a minimum degree of $CD8^+$ T-cell infiltration. This corresponds to the experimental mean relative number of effectors 0.05 minus the standard deviation 0.025 in the follicular phase. We then consider that the appearance and disappearance rates of $CD8^+$ T-lymphocytes are respectively modulated by the chemoattractant signal from dying epithelial cells as follows

$$r_A\left(1+\frac{C_A^2}{\gamma+C_A^2}\right)$$

and

$$r_D\left(1-\frac{C_A^2}{\gamma+C_A^2}\right),$$

where $C_A(x)$ is the concentration of chemokines from dying epithelial cells in x. The parameters $r_D = 0.2$ and $\gamma = 0.02$ were calibrated to reproduce the immune dynamics during the menstrual cycle, compare Fig. 3a-c (experimental data) with Fig. 5 (model results).

Our experimental observations also evidence that the amount of immune cells in the interlobular stroma is significantly low compared to the intralobular stroma. Lymphocytes are present and mainly localized within lobules rather than interlobular stroma, with T-cells directly integrated in the lobular epithelium as part of the immune system (Figs. S8, S10 and S11).[41] Accordingly, we assume that the relative number of immune cells in the interlobular stroma is equal to $\left(\overline{N}_{\text{reg}}+\overline{N}_{\text{eff}}\right)/10$, which is in agreement with our experimental estimates.

In addition, chemokines from damaged epithelial cells modulate the trafficking of both regulatory and effector cells as follows

$$r_A\left(1+\frac{C_A^2}{\gamma+C_A^2}\right)\cdot\left(1+\frac{C_D^2}{\gamma+C_D^2}\right) \tag{3}$$

and

$$r_D\left(1-\frac{C_A^2}{\gamma+C_A^2}\right)\cdot\left(1-\frac{C_D^2}{\gamma+C_D^2}\right), \tag{4}$$

where $C_D(x)$ is the concentration of chemokines from damaged epithelial cells in x. The expressions above are considered for the trafficking of effector cells. However, $C_A(x)$ is always considered equal to zero in the case of regulatory cells. This means that their trafficking is only affected by chemokines from damaged epithelial cells.

At the end of each simulation time-step, immune cell populations are updated through an iterative process. Each inactivated immune cell can leave the system at a rate given by Equation (4). In turn, iterating separately over the nodes of each cross-section of TDLUs and the intralobular stroma, immune cells can appear at a rate given by Equation (3). In this case, a random number is generated to avoid any order bias in the appearance of regulatory and effector cells. In particular, we asume that effector and regulatory cells can enter in the system at the same probability.

Mechanistic rules governing the immune cell dynamics

Fig. S4 provides a schematic representation of the mechanistic rules governing the immune cell dynamics considered in the model, which are summarized in the following:

(1) Immune cells become activated by chemokines from damaged epithelial cells.

(2) Activated immune cells follow the chemoattractant signal (chemotactic movement) from damaged epithelial cells.

(3-4) Activated immune cells become inactive in the absence of the chemoattractant signal from damaged epithelial cells.

(5) Inactivated immune cells follow the chemoattractant signal (chemotactic movement) from dying epithelial cells.

(6) Inactivated immune cells move randomly in the absence of chemoattractant signals.

(7) Activated regulatory cells can permanently inactivate, by direct contact, effector cells.

(8) Activated effector cells can induce death, by direct contact, to damaged epithelial cells.

(9) Trafficking of immune cells is assumed through the blood vessels and modulated by chemokines from dying and/or damaged epithelial cells.

Distribution of patients with respect to the menstrual cycle phase and length

Menstrual cycle length varies from 25 to 38 days, with 10 out of 22 patients having a cycle length of 28 days (Tab. S1). The menstrual cycle phases were determined by anamnestic documentation of the menses prior to the surgery. This was complemented by asking the patients about the date of onset of the first menstruation after operation (Tab. S2).

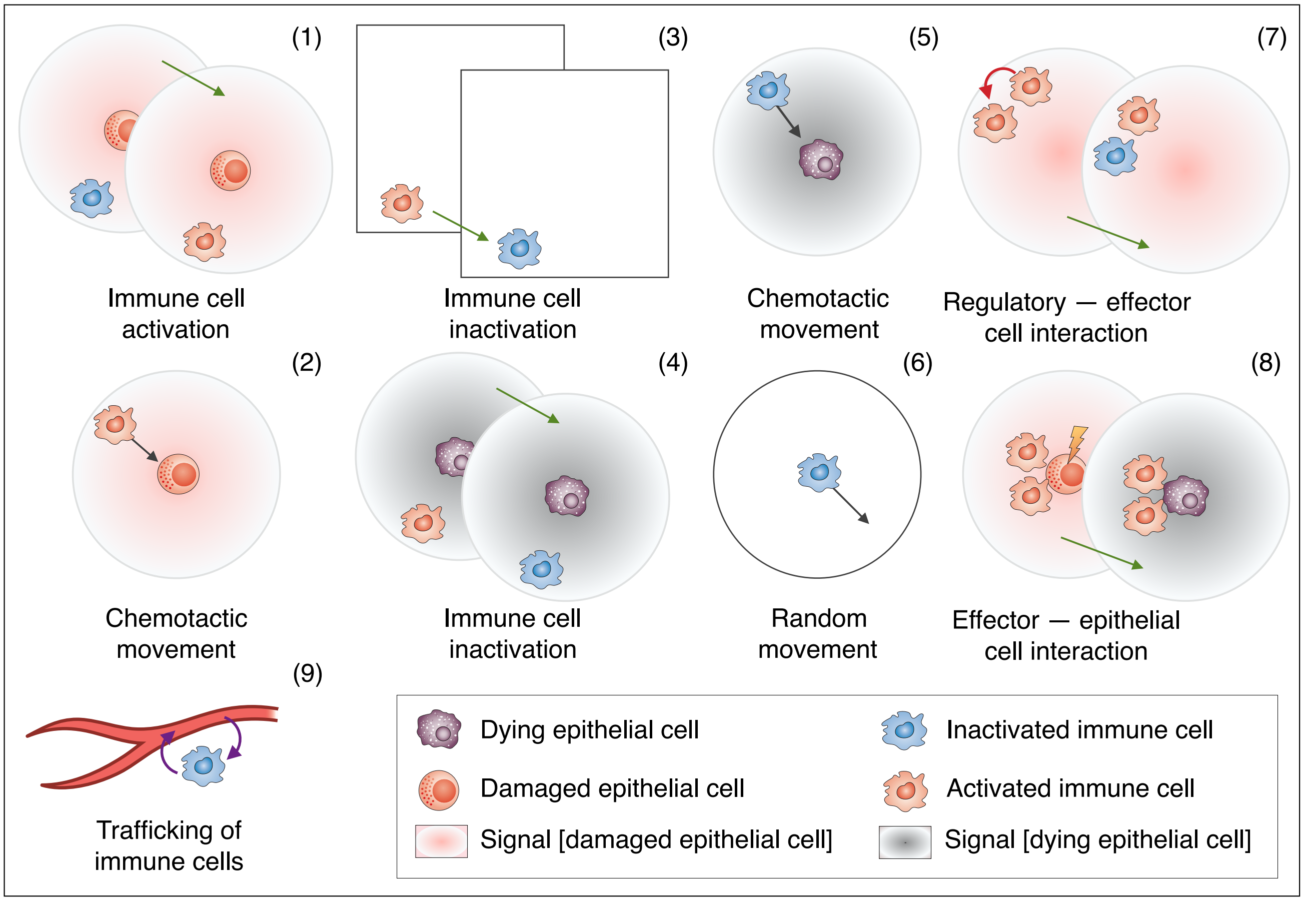

Figure S4. **Mechanistic rules governing the immune cell dynamics**.

Spatial distribution of immune cells in breast tissue

Fig. S5a,c shows that proliferating cells (Ki-67) are uniformly distributed in normal breast tissue, sometimes with slightly increased frequencies at the transition between ducts and lobular epithelium. This is in line with our observation that immune cells are also evenly distributed in the lobular tissue (Figs. S5e,f, S8, S10 and S11). The frequency of positive cells in our series is consistent with published data.[31] We also observe in Fig. S5b,d that only few cells are stained by the cleaved caspase-3 marker for activation of a specific apoptosis pathway. Expectedly, their overall amount is lower than the number of Ki-67 positive cells since only short phases within the time course of apoptosis are labeled, whereas Ki-67 stains all cycle phases in preparation and after mitoses except the resting cells in G0 phase. In particular, we worked under the assumption of an overall balance between cell proliferation and death, considering that the mature breast is usually not undergoing significant changes in size in adult life.

As shown in Fig.3a, we found impressingly high amounts of CD8 positive cytotoxic T-cells in normal breast tissue (Fig. S5e,g), which is consistent with published data.[41] A double staining of collagen IV and CD3 T-cells in Fig. S5f,h shows that the majority of T-cells are located within the epithelial lining, often close to the luminal side of the basal lamina. Fig. S6 provides experimental data about the relative location of $CD8^+$, $CD163^+$ and $CD4^+$ cells in normal lobular epithelium. The terms *contact*, *close* and *stroma* mean (i) direct contact with at least one epithelial cell, (ii) at most two-cell diameter distance from any epithelial cell and (iii) in the intralobular stroma, respectively. Fig. S7 shows the model predicted time evolutions of the relative number of immune cells with respect to location in the lobular tissue. We obtain that calibrated model parameters provide accurate estimates of the relative number and spatial distribution of effector and regulatory cells during the menstrual cycle, compare Fig. S6 (experimental data) to Fig. S7 (model results).

Figs. S8, S9, S10 and S11 show immunohistochemical stainings for CD8 (effector cells), Ki-67 (proliferating epithelial cells), CD163 (macrophages) and CD4 (T-regulatory cells) in breast lobular tissue from healthy women with respect to the

Menstrual Cycle Length (days)	Number of Patients
25	1
26	2
27	2
28	10
30	3
31	1
34	2
38	1

Table S1. Distribution of the menstrual cycle length in the cohort of patients considered.

Follicular Phase (days)	Number of Patients	Luteal Phase (days)	Number of Patients
30	1	11	3
28	1	8	3
27	1	7	3
25	1	5	2
22	2	4	1
21	1	3	2
−−	−−	2	1

Table S2. Distribution of the menstrual cycle phases in the cohort of patients considered.

menstrual cycle phase. In the cases of CD8 and Ki-67 more positive cells are commonly seen in the luteal than follicular phase of the menstrual cycle. However, the amount of CD163^{+} and CD4^{+} cells remains almost invariant during the menstrual cycle (Fig. S6).

Fig. S12 provides experimental data about the relative location of CD8^{+}, CD163^{+} and CD4^{+} cells in lobular tissue from women who underwent reduction mammoplasty (RM) due to orthopedic or cosmetic reasons, and prophylactic mastectomy (PM) due to *BRCA1/2* mutations. Moreover, the relative location of immune cells in breast cancer/neoplastic tissue (NT) samples is also provided.

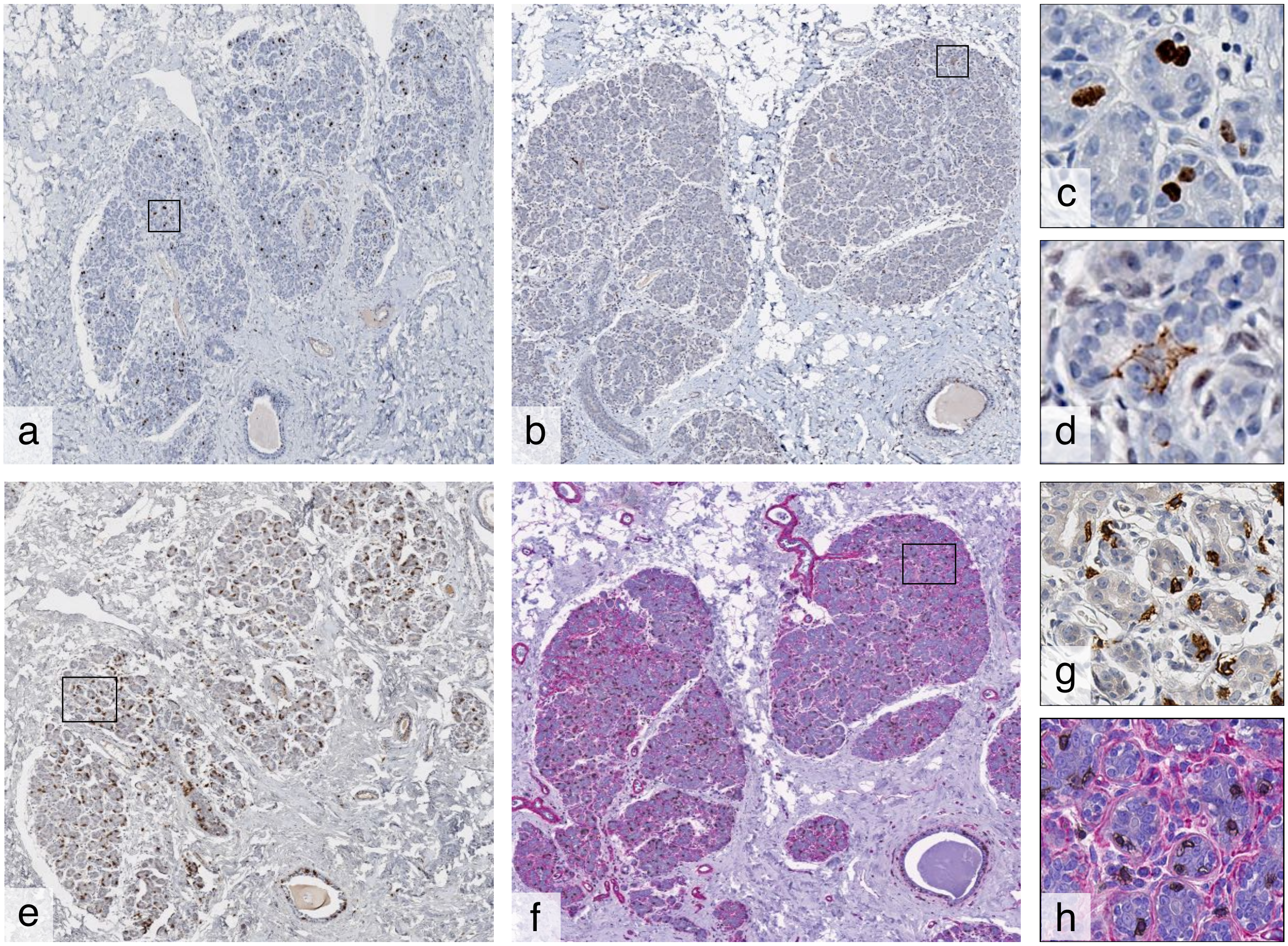

Figure S5. Spatial distribution of immune cells and cellular events in breast tissue from a healthy woman. Representative regions showing **(a,c)** proliferating cells [Ki-67], **(b,d)** apoptotic cells [cleaved caspase-3], **(e,g)** cytotoxic T-cells [CD8], and **(f,h)** T-cells in general in relation to the basal lamina [double staining Collagen IV (red) and CD3 (brown)]. The breast tissue samples correspond to a 28-year-old premenopausal woman without clinical abnormality nor familial history of breast cancer, and in the luteal phase of the menstrual cycle (28 days long) at the time of the biopsy.

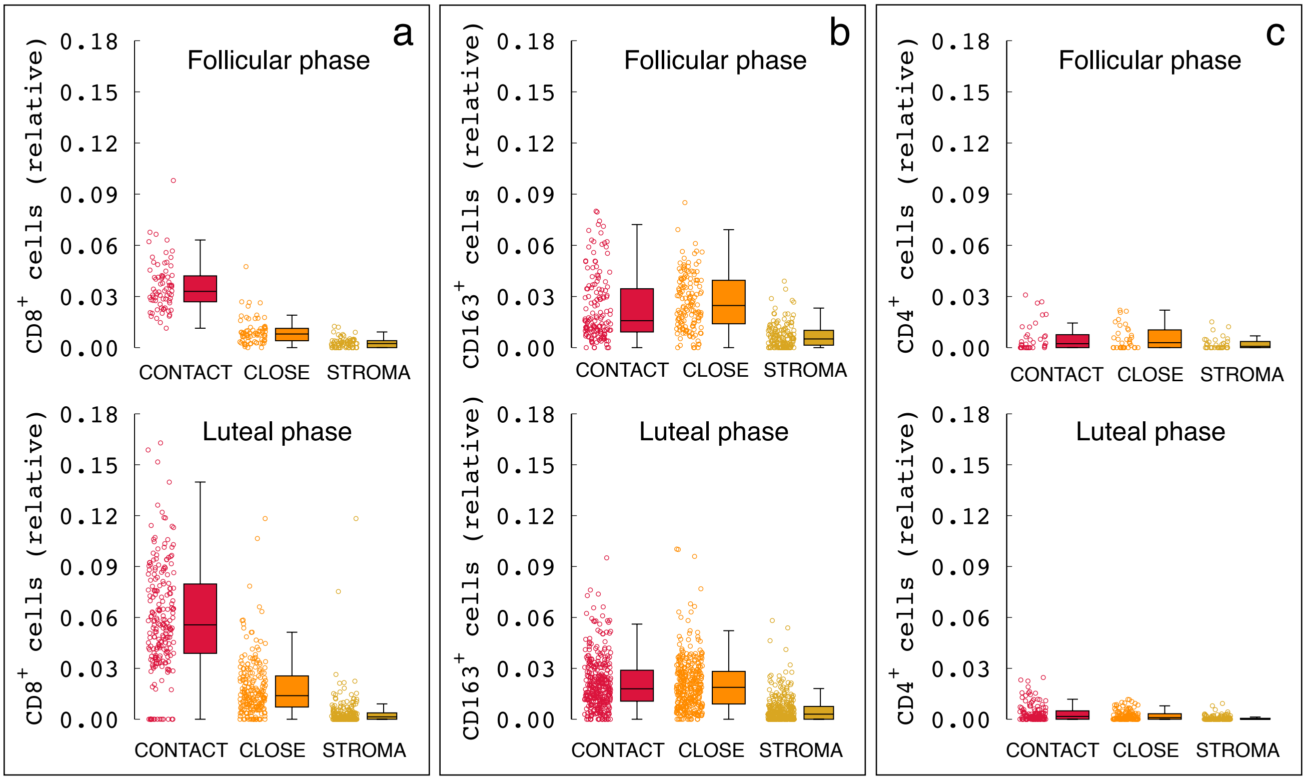

Figure S6. Spatial distribution of immune cells in normal breast tissue. Relative number of **(a)** CD8$^+$, **(b)** CD163$^+$ and **(c)** CD4$^+$ cells with respect to the menstrual cycle phase and the location in the lobular epithelium, i.e. (*contact*, *close* and (intralobular) *stroma*). Each box is drawn around the region between the first and third quartiles of the data points, with a horizontal line at the median value and whiskers extend for a range equal to 1.5 times the interquartile range.

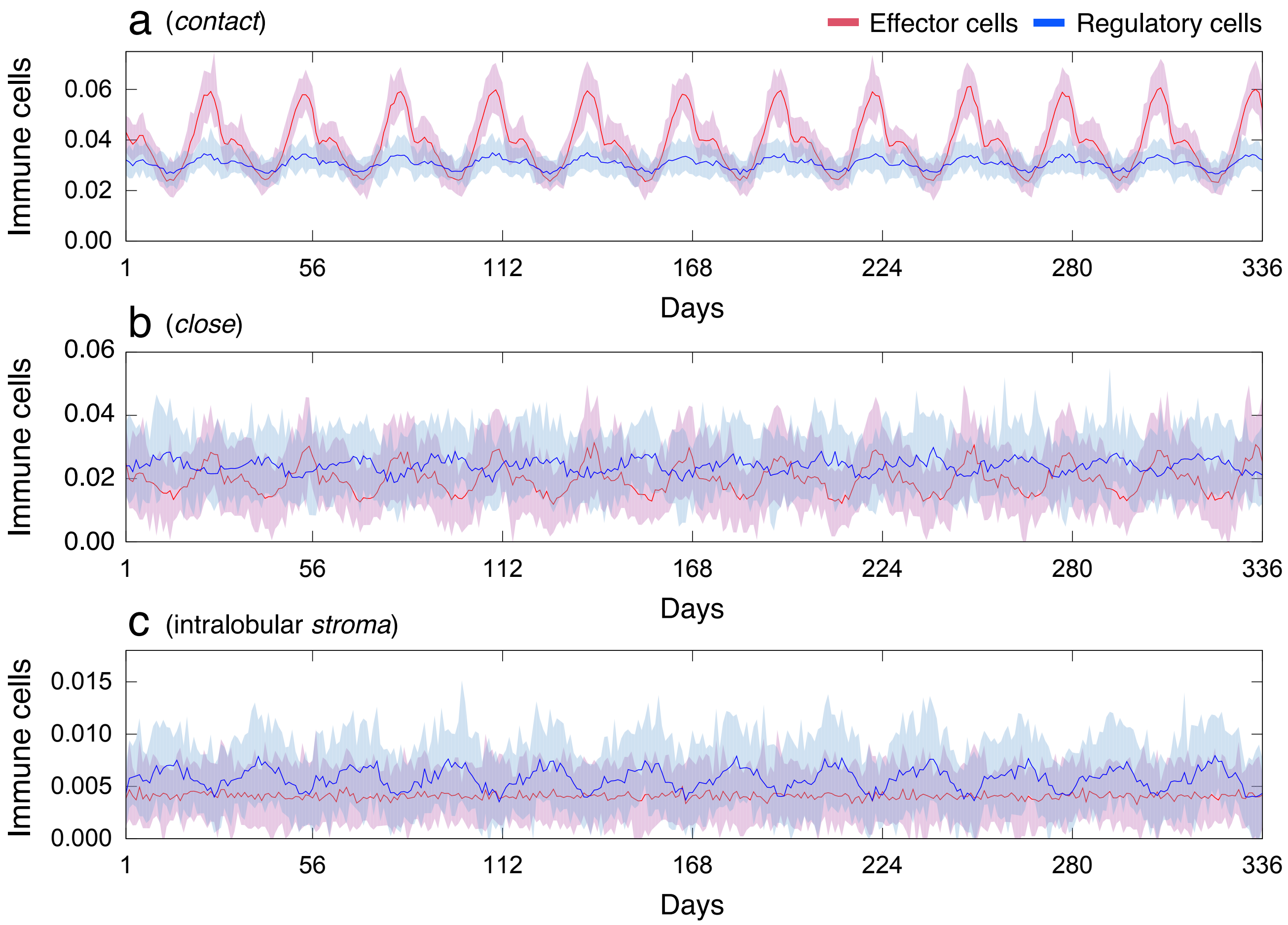

Figure S7. Time evolution and spatial distribution of immune cells in healthy breast tissue. The results are averaged over 20 simulations each consisting in 12 menstrual cycles of 28 days and normal hormone levels. The mean (marked solid line) and min/max (shadow) values are represented. **(a)** Direct contact with at least one epithelial cell (*contact*), **(b)** at most two-cell diameter distance from any epithelial cell (*close*) and **(c)** in the intralobular (*stroma*).

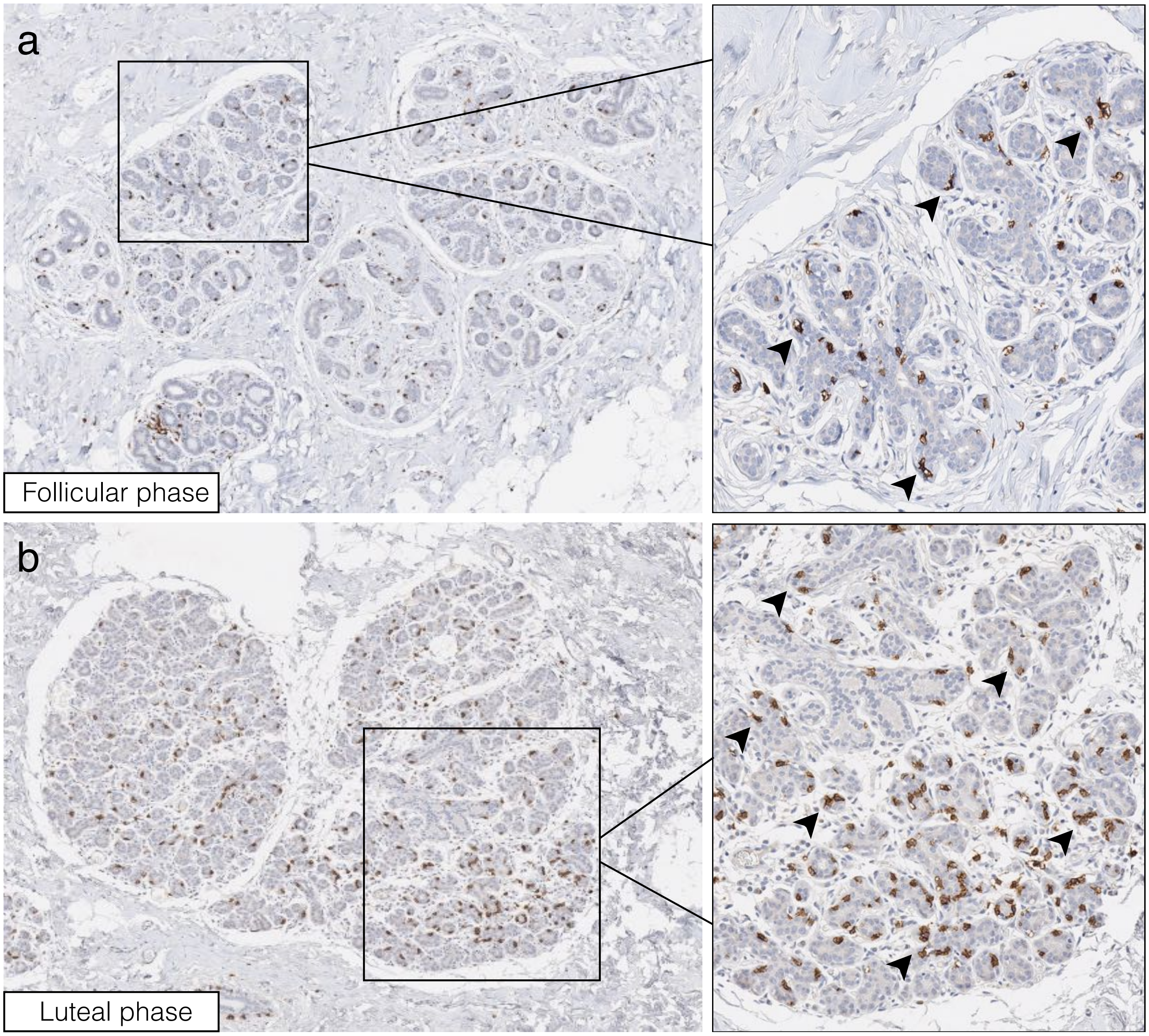

Figure S8. Immunohistochemical stainings for CD8 (effector cells) in breast lobular tissue from different healthy women. **(a)** Follicular and **(b)** luteal phases of the menstrual cycle at the time of the biopsies. The breast tissue samples correspond to premenopausal women (27- and 28-year-old) without clinical abnormality nor familial history of breast cancer, and with a menstrual cycle length of 28 days.

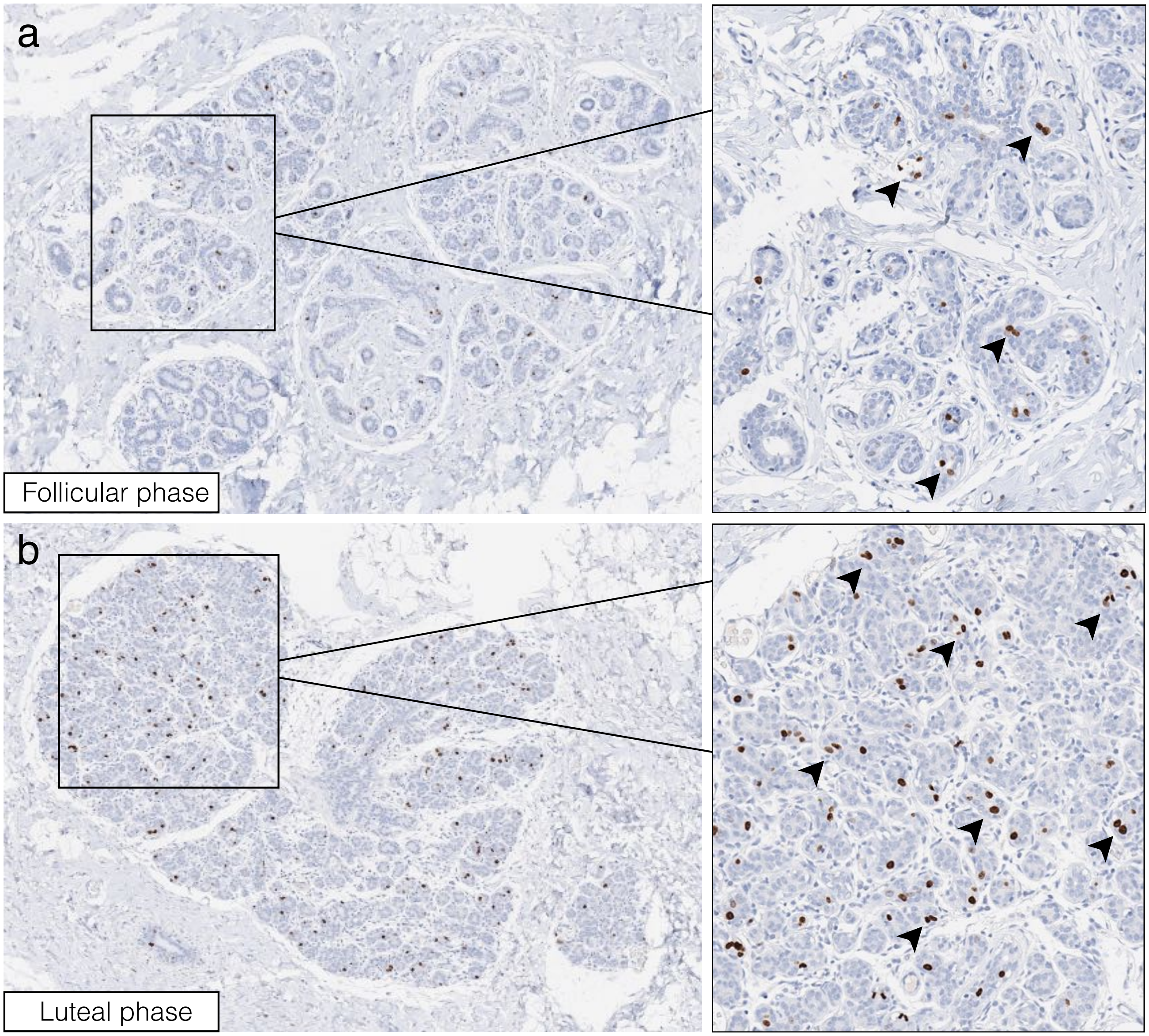

Figure S9. **Immunohistochemical stainings for Ki-67 (proliferating epithelial cells) in breast lobular tissue from different healthy women**. **(a)** Follicular and **(b)** luteal phases of the menstrual cycle at the time of the biopsies. The breast tissue samples correspond to premenopausal women (27- and 28-year-old) without clinical abnormality nor familial history of breast cancer, and with a menstrual cycle length of 28 days.

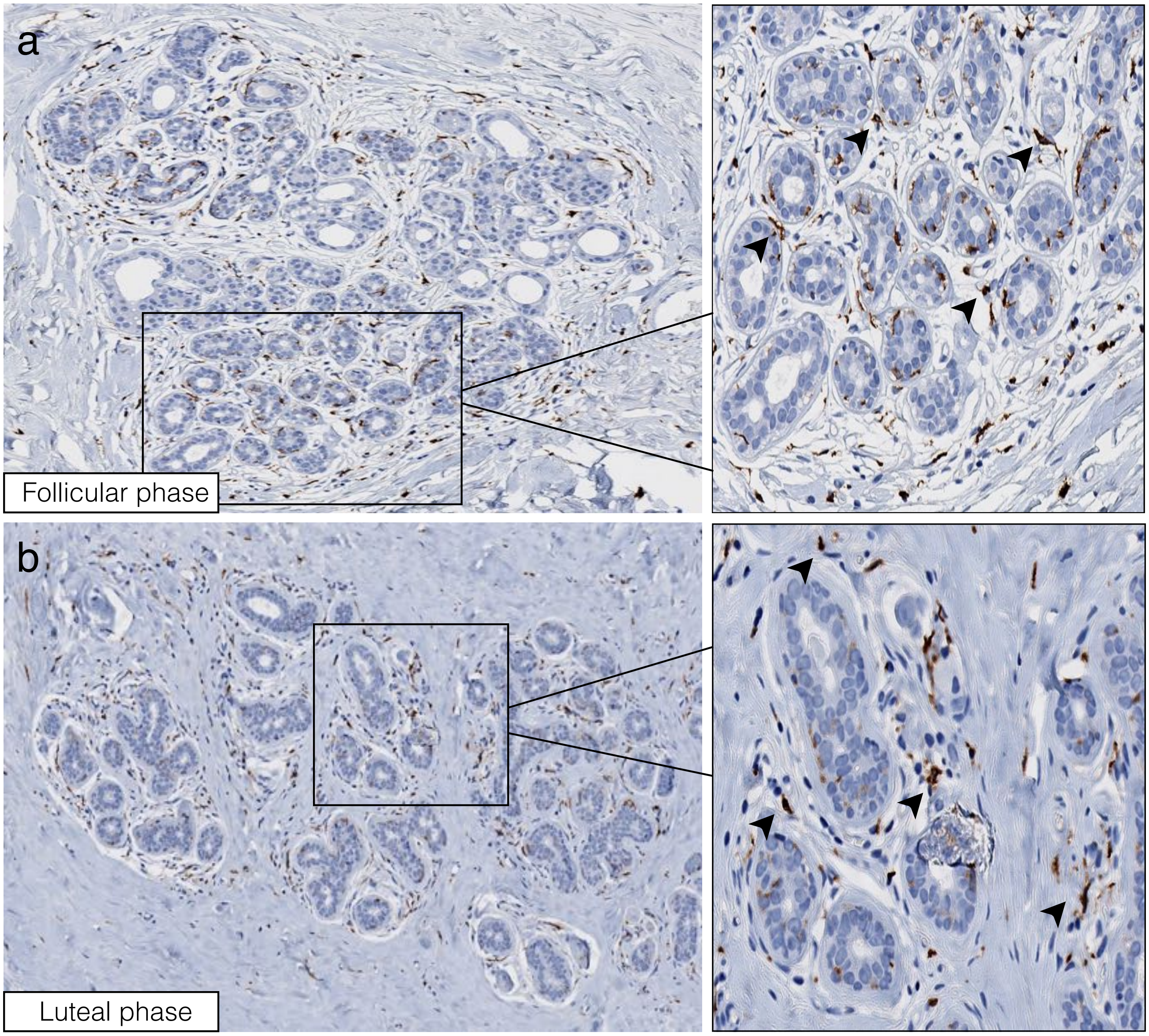

Figure S10. Immunohistochemical stainings for CD163 (macrophages) in breast lobular tissue from different healthy women. **(a)** Follicular and **(b)** luteal phases of the menstrual cycle at the time of the biopsies. The breast tissue samples correspond to premenopausal women (29- and 20-year-old) without clinical abnormality nor familial history of breast cancer, and with menstrual cycle lengths of 27 and 28 days.

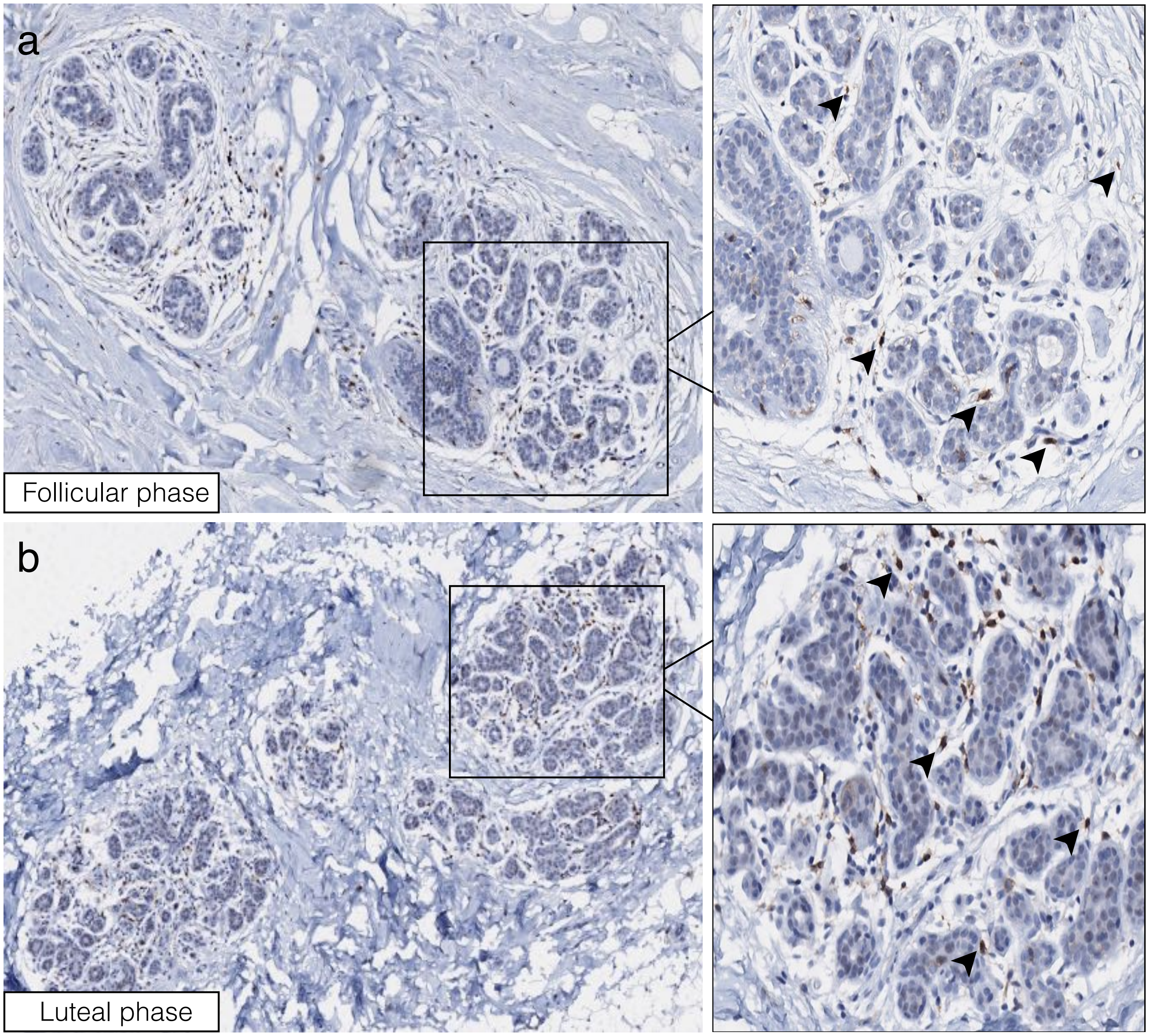

Figure S11. Immunohistochemical stainings for CD4 (T-regulatory cells) in breast lobular tissue from different healthy women. **(a)** Follicular and **(b)** luteal phases of the menstrual cycle at the time of the biopsies. The breast tissue samples correspond to premenopausal women (29- and 25-year-old) without clinical abnormality nor familial history of breast cancer, and with menstrual cycle lengths of 27 and 30 days.

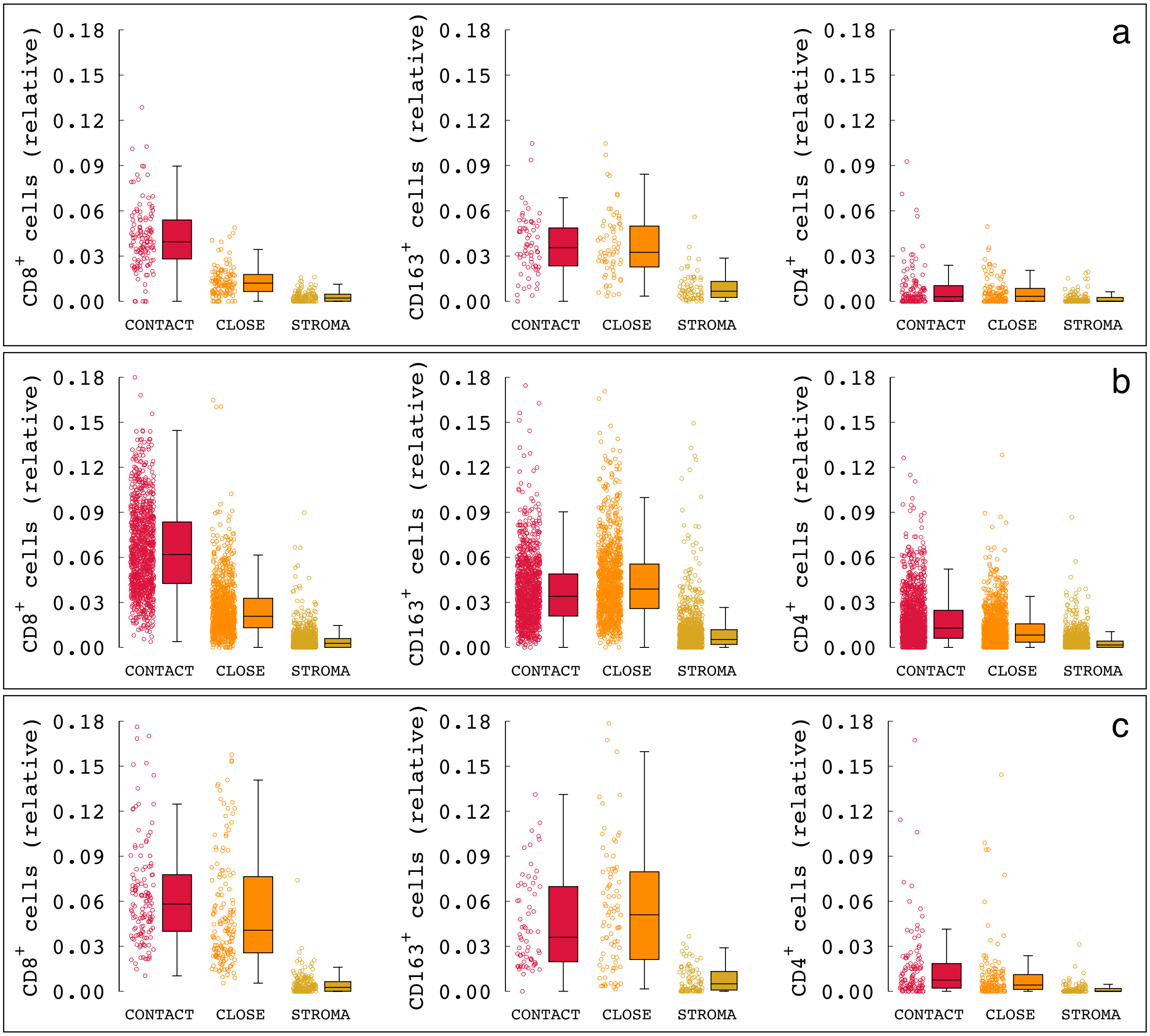

Figure S12. **Spatial distribution of immune cells in normal and neoplastic breast tissue**. Quantification of immune infiltrates in breast lobular tissue of women who underwent **(a)** reduction mammoplasty (RM) due to orthopedic or cosmetic reasons, and **(b)** prophylactic mastectomy (PM) due to *BRCA1/2* mutations. Amount of immune cells in **(c)** normal lobular tissue adjacent (more than 1 mm) to breast cancer/neoplastic tissue (NT) is also quantified. From left to right, relative number of $CD8^+$, $CD163^+$ and $CD4^+$ cells with respect to the location in the lobular epithelium, i.e. (*contact*, *close* and (intralobular) *stroma*). Each box is drawn around the region between the first and third quartiles of the data points, with a horizontal line at the median value and whiskers extend for a range equal to 1.5 times the interquartile range.

References

1. Russo, J., Ao, X., Grill, C. & Russo, I. Pattern of distribution of cells positive for estrogen receptor α and progesterone receptor in relation to proliferating cells in the mammary gland. *Breast Cancer Research and Treatment* **53**, 217–227 (1999).
2. Russo, J. & Russo, I. H. Histological evaluation of the normal breast. In *Techniques and Methodological Approaches in Breast Cancer Research*, 45–73 (Springer, 2014).
3. Rejniak, K. A. *et al.* Linking changes in epithelial morphogenesis to cancer mutations using computational modeling. *PLoS Comput. Biol* **6**, e10009000 (2010).
4. Pommerville, J. *et al. Alcamo's fundamentals of microbiology: Body systems* (Jones & Bartlett Publishers, 2012).
5. Baggiolini, M. Chemokines and leukocyte traffic. *Nature* **392**, 565–568 (1998).
6. Moser, B. & Loetscher, P. Lymphocyte traffic control by chemokines. *Nature Immunology* **2**, 123–128 (2001).
7. Thelen, M. & Stein, J. V. How chemokines invite leukocytes to dance. *Nature Immunology* **9**, 953–959 (2008).
8. Esche, C., Stellato, C. & Beck, L. A. Chemokines: key players in innate and adaptive immunity. *Journal of Investigative Dermatology* **125**, 615–628 (2005).
9. Janeway, C. A., Travers, P., Walport, M., Shlomchik, M. J. *et al. Immunobiology: the immune system in health and disease*, vol. 2 (Garland New York, 2001).
10. Matzavinos, A., Chaplain, M. A. & Kuznetsov, V. A. Mathematical modelling of the spatio-temporal response of cytotoxic t-lymphocytes to a solid tumour. *Mathematical Medicine and Biology* **21**, 1–34 (2004).
11. Chaplain, M. A. The mathematical modelling of tumour angiogenesis and invasion. *Acta Biotheoretica* **43**, 387–402 (1995).
12. Gammack, D., Doering, C. & Kirschner, D. Macrophage response to mycobacteriumtuberculosis infection. *Journal of mathematical biology* **48**, 218–242 (2004).
13. Chaplain, M. A. & Lolas, G. Mathematical modelling of cancer invasion of tissue: dynamic heterogeneity. *NHM* **1**, 399–439 (2006).
14. Fallahi-Sichani, M., Kirschner, D. E. & Linderman, J. J. Nf-κb signaling dynamics play a key role in infection control in tuberculosis. *Frontiers in Physiology* **3** (2012).
15. Hillyer, P. & Male, D. Expression of chemokines on the surface of different human endothelia. *Immunology and Cell Biology* **83**, 375–382 (2005).
16. Hamilton, T. A. *et al.* Chemokine and chemoattractant receptor expression: post-transcriptional regulation. *Journal of Leukocyte Biology* **82**, 213–219 (2007).
17. Grasselli, M. & Pelinovsky, D. *Numerical mathematics* (Jones & Bartlett Learning, 2008).
18. Thomas, J. W. *Numerical partial differential equations: finite difference methods*, vol. 22 (Springer Science & Business Media, 2013).
19. Miller, M. J., Wei, S. H., Parker, I. & Cahalan, M. D. Two-photon imaging of lymphocyte motility and antigen response in intact lymph node. *Science* **296**, 1869–1873 (2002).
20. Mueller, S. N. Effector t-cell responses in non-lymphoid tissues: insights from in vivo imaging. *Immunology and Cell Biology* **91**, 290–296 (2013).
21. Masopust, D. & Schenkel, J. M. The integration of t cell migration, differentiation and function. *Nature Reviews Immunology* **13**, 309–320 (2013).
22. Pechoux, C., Gudjonsson, T., Rønnov-Jessen, L., Bissell, M. J. & Petersen, O. W. Human mammary luminal epithelial cells contain progenitors to myoepithelial cells. *Developmental Biology* **206**, 88–99 (1999).
23. Ramakrishnan, R., Khan, S. A. & Badve, S. Morphological changes in breast tissue with menstrual cycle. *Modern Pathology* **15**, 1348–1356 (2002).
24. Smith, G. Mammary epithelial stem cells. In Sell, S. (ed.) *Stem Cells Handbook*, 437–443 (Humana Press, 2004).
25. Gudjonsson, T., Adriance, M. C., Sternlicht, M. D., Petersen, O. W. & Bissell, M. J. Myoepithelial cells: their origin and function in breast morphogenesis and neoplasia. *Journal of Mammary Gland biology and Neoplasia* **10**, 261–272 (2005).

26. Russo, J. & Russo, I. H. Influence of differentiation and cell kinetics on the susceptibility of the rat mammary gland to carcinogenesis. *Cancer Research* **40**, 2677–2687 (1980).

27. Russo, I. H. & Russo, J. Mammary gland neoplasia in long-term rodent studies. *Environmental Health Perspectives* **104**, 938 (1996).

28. Mukhopadhyay, R. *et al.* Promotion of variant human mammary epithelial cell outgrowth by ionizing radiation: an agent-based model supported by in vitro studies. *Breast Cancer Research* **12**, R11 (2010).

29. Band, V. & Sager, R. Distinctive traits of normal and tumor-derived human mammary epithelial cells expressed in a medium that supports long-term growth of both cell types. *Proceedings of the National Academy of Sciences* **86**, 1249–1253 (1989).

30. Stampfer, M. R. & Bartley, J. C. Induction of transformation and continuous cell lines from normal human mammary epithelial cells after exposure to benzo [a] pyrene. *Proceedings of the National Academy of Sciences* **82**, 2394–2398 (1985).

31. Navarrete, M. *et al.* Assessment of the proliferative, apoptotic and cellular renovation indices of the human mammary epithelium during the follicular and luteal phases of the menstrual cycle. *Breast Cancer Research* **7**, R306–13 (2005).

32. Van Cruchten, S. & Van Den Broeck, W. Morphological and biochemical aspects of apoptosis, oncosis and necrosis. *Anatomia, Histologia, Embryologia* **31**, 214–223 (2002).

33. Bursch, W., Kleine, L. & Tenniswood, M. The biochemistry of cell death by apoptosis. *Biochemistry and Cell Biology* **68**, 1071–1074 (1990).

34. Goergen, J., Marc, A. & Engasser, J. Determination of cell lysis and death kinetics in continuous hybridoma cultures from the measurement of lactate dehydrogenase release. *Cytotechnology* **11**, 189–195 (1993).

35. Wells, J. E. & Russell, J. B. The effect of growth and starvation on the lysis of the ruminal cellulolytic bacterium fibrobacter succinogenes. *Applied and Environmental Microbiology* **62**, 1342–1346 (1996).

36. Trapani, J. A. & Smyth, M. J. Functional significance of the perforin/granzyme cell death pathway. *Nature Reviews Immunology* **2**, 735–747 (2002).

37. Stein, J. V. & Nombela-Arrieta, C. Chemokine control of lymphocyte trafficking: a general overview. *Immunology* **116**, 1–12 (2005).

38. Strell, C. & Entschladen, F. Extravasation of leukocytes in comparison to tumor cells. *Cell Communication and Signaling* **6**, 1 (2008).

39. Gjorevski, N. & Nelson, C. M. Integrated morphodynamic signalling of the mammary gland. *Nature Reviews Molecular Cell Biology* **12**, 581–593 (2011).

40. Brisken, C. Progesterone signalling in breast cancer: a neglected hormone coming into the limelight. *Nature Reviews Cancer* **13**, 385–396 (2013).

41. Degnim, A. C. *et al.* Immune cell quantitation in normal breast tissue lobules with and without lobulitis. *Breast Cancer Research and Treatment* **144**, 539–549 (2014).
